# Common genetic variations in cell cycle and DNA repair pathways associated with pediatric brain tumor susceptibility

**DOI:** 10.18632/oncotarget.11575

**Published:** 2016-08-24

**Authors:** Maral Adel Fahmideh, Catharina Lavebratt, Joachim Schüz, Martin Röösli, Tore Tynes, Michael A. Grotzer, Christoffer Johansen, Claudia E Kuehni, Birgitta Lannering, Michaela Prochazka, Lisbeth S Schmidt, Maria Feychting

**Affiliations:** ^1^ Unit of Epidemiology, Institute of Environmental Medicine, Karolinska Institutet, SE-171 77 Stockholm, Sweden; ^2^ Neurogenetics Unit, Department of Molecular Medicine and Surgery, Karolinska Institutet, and Center for Molecular Medicine, Karolinska University Hospital, L8:00, SE-171 76 Stockholm, Sweden; ^3^ Section of Environment and Radiation, International Agency for Research on Cancer (IARC), 69372 Lyon, France; ^4^ Department of Epidemiology and Public Health, Swiss Tropical and Public Health Institute, 4002 Basel, Switzerland; ^5^ University of Basel, 4003 Basel, Switzerland; ^6^ The Cancer Registry of Norway, NO-0379 Oslo, Norway; ^7^ National Institute of Occupational Health, NO-0360 Oslo, Norway; ^8^ Department of Oncology, University Children's Hospital of Zurich, 8032 Zurich, Switzerland; ^9^ Unit of Survivorship, The Danish Cancer Society Research Centre, DK-2100 Copenhagen, Denmark; ^10^ Oncology Department, Finsen Centre, Rigshospitalet, DK-2100 Copenhagen, Denmark; ^11^ Swiss Childhood Cancer Registry, Institute of Social and Preventive Medicine, University of Bern, 3012 Bern, Switzerland; ^12^ Childrens Cancer Center, Queen Silvia Childrens Hospital, SE-416 85 Gothenburg, Sweden; ^13^ Department of Clinical Genetics, University Hospital Rigshospitalet, DK-2100 Copenhagen, Denmark

**Keywords:** genetic association study, pediatric brain tumors, single nucleotide polymorphism, brain neoplasm, susceptibility

## Abstract

Knowledge on the role of genetic polymorphisms in the etiology of pediatric brain tumors (PBTs) is limited. Therefore, we investigated the association between single nucleotide polymorphisms (SNPs), identified by candidate gene-association studies on adult brain tumors, and PBT risk.

The study is based on the largest series of PBT cases to date. Saliva DNA from 245 cases and 489 controls, aged 7–19 years at diagnosis/reference date, was genotyped for 68 SNPs. Data were analyzed using unconditional logistic regression.

The results showed *EGFR*rs730437 and *EGFR*rs11506105 may decrease susceptibility to PBTs, whereas *ERCC1*rs3212986 may increase risk of these tumors. Moreover, stratified analyses indicated *CHAF1A*rs243341, *CHAF1A*rs2992, and *XRCC1*rs25487 were associated with a decreased risk of astrocytoma subtype. Furthermore, an increased risk of non-astrocytoma subtype associated with *EGFR*rs9642393, *EME1*rs12450550, *ATM*rs170548, and *GLTSCR*rs1035938 as well as a decreased risk of this subtype associated with *XRCC4*rs7721416 and *XRCC4*rs2662242 were detected.

This study indicates SNPs in *EGFR*, *ERCC1*, *CHAF1A*, *XRCC1*, *EME1*, *ATM*, *GLTSCR1*, and *XRCC4* may be associated with the risk of PBTs. Therefore, cell cycle and DNA repair pathways variations associated with susceptibility to adult brain tumors also seem to be associated with PBT risk, suggesting pediatric and adult brain tumors might share similar etiological pathways.

## INTRODUCTION

Brain tumors are the most common pediatric solid tumors and the leading cause of cancer mortality in children. High-dose ionizing radiation and rare inherited syndromes are the only established risk factors for brain tumors, causing a small proportion of cases [1]. Thus, brain tumors are considered as multifactorial disorders resulting from progressive accumulation of genetic and epigenetic alterations in concert with environmental exposures. It has been suggested that genetic polymorphisms in four main pathways including DNA repair, cell cycle, metabolism, and inflammation play an important role in brain carcinogenesis [2]. Therefore, candidate gene-association studies of adult brain tumors have mainly focused on these four hypothesized pathways to identify genetic susceptibility factors for adult brain tumors [3–9].

Whereas a considerable number of candidate gene-association studies are available on adult brain tumors, very few and small genetic studies have been performed on brain tumors in children and adolescents [10–13], due to difficulties in collecting a sufficient number of DNA samples. Hence, the role of genetic polymorphisms in pediatric brain tumor (PBT) etiology is largely unknown. However, some studies have found similar genetic mutation patterns for adult and pediatric brain tumor progression within specific histological types [14–18]. These similarities in prognostic factors provide an important starting point for identifying candidate genetic risk factors for PBTs; that might be similar to those involved in adult brain tumorigenesis [19]. Moreover, in our previous genetic association study of PBT risk, we concluded that single nucleotide polymorphisms (SNPs) identified by genome-wide association studies (GWAS) on adult glioma might also be associated with the risk of brain tumors in children [20].

The aim of this study, which represents the largest series of PBT cases to date, was to determine whether the SNPs identified by candidate gene-association studies on adult brain tumors, involved in the four main pathways discussed above, are also related to pediatric brain tumor risk.

## RESULTS

We successfully genotyped 63 SNPs in 245 cases and 489 controls. The distributions of allele frequencies in 3 SNPs (rs4444903, rs9288516, and rs61754966) were not in agreement with HWE (*p* < 0.001) and therefore these SNPs were dropped from analyses. As shown in Table 1, the age and sex distributions were similar in cases and controls.

**Table 1:** Characteristics of cases and controls

Characteristics	Cases	Astrocytomas	Non-astrocytomas	Controls
**No. of participants**	245	134	111	489
**Sex**				
Males	136 (56%)	74 (55%)	62 (56%)	261 (53%)
Females	109 (44%)	60 (45%)	49 (44%)	228 (47%)
**Age-group (at diagnosis/reference date)**				
7–9 years old	48 (20%)	28 (21%)	20 (18%)	112 (23%)
10–14 years old	108 (44%)	60 (45%)	48 (43%)	219 (45%)
15–19 years old	89 (36%)	46 (34%)	43 (39%)	158 (32%)
**Country**				
Sweden	106 (43%)	48 (36%)	58 (52%)	174 (36%)
Norway	24 (10%)	15 (11%)	9 (8%)	62 (13%)
Denmark	62 (25%)	37 (28%)	25 (23%)	134 (27%)
Switzerland	53 (22%)	34 (25%)	19 (17%)	119(24%)
**Type of tumor (ICCC-3 group III)**^a^				
Astrocytoma (IIIb)	134 (55%)			
Pilocytic astrocytoma	93			
Supependymal giant cell astrocytoma	5			
Pleomorphic xanthoastrocytoma	4			
Diffuse astrocytoma	13			
Anaplastic astrocytoma	11			
Fibrillary astrocytoma	2			
Glioblastoma	5			
Giant cell glioblastoma	1			
Other gliomas (IIId)	20 (8%)			
Malignant glioma	11			
Oligoastrocytoma	2			
Oligodendroglioma	6			
Anaplastic oligodendroglioma	1			
Ependymoma (IIIa)	19 (8%)			
Subependymoma	2			
Choroid plexus papilloma	4			
Choroid plexus caricinoma	1			
Ependymoma	7			
Papillary ependymoma	1			
Anaplastic ependymoma	4			
Intracranial embryonal tumors (IIIc)	7 (3%)			
CNS primitive neuroectodermal tumor	6			
Neuroepithelioma	1			
Other specified intracranial neoplasms (IIIe)	49 (20%)			
Germinoma	7			
Yolk sac tumor	1			
Teratoma, mature	1			
Haemangioblastoma	1			
Desmoplastic infantile ganglioglioma	2			
Dysembryoplastic neuroepithelial tumor	6			
Ganglioglioma	26			
Anaplastic ganglioglioma	1			
Centrol neurocytoma	3			
Neurilemoma	1			
Unspecified intracranial neoplasm (IIIf)	16 (6%)			

a Restricted to ICD-O-3 location C71, subclassified according to WHO histological subclassification, 2007; patients with neurofibromatosis and tuberous sclerosis were excluded.

As Table 2 illustrates, the A alleles of *EGFR* rs730437 (OR_DOM_ 0.59 [95% CI 0.42-0.83], *p* = 0.002) and *EGFR* rs11506105 (OR_DOM_ 0.71 [95% CI 0.51–0.98], *p* = 0.036) involved in cell cycle pathway were associated with decreased susceptibility to PBTs, whereas the A allele of *ERCC1* rs3212986 (OR_DOM_ 1.53 [95% CI 1.11–2.09], *p* = 0.009) involved in DNA repair pathway was associated with an increased risk of these tumors. Moreover, the interactions between these SNPs and covariates including age, sex and country were not significant.

**Table 2:** Summary results for SNPs associated with pediatric brain tumors

SNP	Chr.	Gene	Pathway	Location (bp)	Minor allele	MAF^a^ in cases	MAF^a^ in controls	Model	OR^b^	95% CI	*P*	CHISQ	Pinter^c^
rs730437	7	*EGFR*	Cell cycle	55215018	A	0.44	0.51	Dominant	0.59	0.42–0.83	0.002		0.435
								Recessive	0.79	0.56–1.15	0.23		
								Additive	0.75	0.60–0.93	0.009		
								Allelic			0.016	5.77	
rs11506105	7	*EGFR*	Cell cycle	55220177	A	0.39	0.45	Dominant	0.71	0.51–0.98	0.036		0.285
								Recessive	0.87	0.59–1.28	0.477		
								Additive	0.82	0.66–1.02	0.079		
								Allelic			0.099	2.73	
rs3212986	19	*ERCC1*	DNA Repair	45912736	A	0.27	0.21	Dominant	1.53	1.11–2.09	0.009		0.311
								Recessive	1.14	0.59–2.19	0.699		
								Additive	1.34	1.04–1.73	0.023		
								Allelic			0.029	4.78	

a MAF=Minor Allele Frequency

b OR adjusted for age, sex, and country

c *P* value for interactions between SNPs and demographic variables including age, sex and country.

The stratified analyses of two histological subtypes indicated that the protective effect of *EGFR* rs730437 remained significant in both patients with astrocytoma and non-astrocytoma tumor subtypes (p_DOM_=0.018 and p_DOM_=0.014, respectively), whereas the risk effect of *ERCC1* rs3212986 was more evident in patients with astrocytoma subtype (p_DOM_=0.002). Moreover, a decreased risk of astrocytoma subtype associated with the C alleles of *CHAF1A* rs243341 and rs2992 as well as the T allele of *XRCC1* rs25487 involved in DNA repair pathway was detected (p_DOM_=0.040, p_DOM_=0.049, and p_DOM_=0.033, respectively). In addition, the stratified analyses showed an increased risk of non-astrocytoma tumor subtype associated with the C alleles of *EGFR* rs9642393, *EME1* rs12450550, and *ATM* rs170548, and the T allele of *GLTSCR1* rs1035938 (p_REC_=0.021, p_REC_=0.001, p_DOM_=0.041, and p_REC_=0.027, respectively) as well as a decreased risk of this subtype associated with the A allele of *XRCC4* rs7721416 and the C allele of *XRCC4* rs2662242 (DNA repair pathway) (p_REC_=0.032 and p_REC_=0.024, respectively) (Tables 3 and 4).

**Table 3:** Summary results for SNPs associated with astrocytoma subtype

SNP	Chr.	Gene	Pathway	Location (bp)	Minor allele	MAF^a^ in cases	MAF^a^ in controls	Model	OR^b^	95% CI	*P*	CHISQ
rs730437	7	*EGFR*	Cell cycle	55215018	A	0.44	0.51	Dominant	0.60	0.39–0.91	0.018	
								Recessive	0.84	0.54–1.33	0.462	
								Additive	0.77	0.59–1.01	0.058	
								Allelic			0.064	3.42
rs3212986	19	*ERCC1*	DNA Repair	45912736	A	0.29	0.21	Dominant	1.87	1.26–2.76	0.002	
								Recessive	0.95	0.40–2.25	0.914	
								Additive	1.49	1.09–2.04	0.011	
								Allelic			0.012	6.36
rs243341	19	*CHAF1A*	DNA Repair	4405106	C	0.23	0.29	Dominant	0.66	0.45–0.98	0.040	
								Recessive	0.62	0.28–1.35	0.228	
								Additive	0.71	0.52–0.98	0.036	
								Allelic			0.033	4.57
rs2992	19	*CHAF1A*	DNA Repair	4443046	C	0.23	0.29	Dominant	0.67	0.45–0.99	0.049	
								Recessive	0.64	0.29–1.39	0.262	
								Additive	0.72	0.53–0.99	0.046	
								Allelic			0.042	4.13
rs25487	19	*XRCC1*	DNA Repair	44055726	T	0.30	0.36	Dominant	0.66	0.44–0.97	0.033	
								Recessive	0.87	0.48–1.59	0.652	
								Additive	0.77	0.57–1.03	0.076	
								Allelic			0.077	3.13

a MAF=Minor Allele Frequency

b OR adjusted for age, sex, and country.

**Table 4:** Summary results for SNPs associated with non-astrocytoma subtype

SNP	Chr.	Gene	Pathway	Location (bp)	Minor allele	MAF^a^ in cases	MAF^a^ in controls	Model	OR^b^	95% CI	*P*	CHISQ
rs730437	7	*EGFR*	Cell cycle	55215018	A	0.44	0.51	Dominant	0.56	0.36–0.89	0.014	
								Recessive	0.75	0.46–1.25	0.271	
								Additive	0.72	0.53–0.97	0.030	
								Allelic			0.060	3.54
rs9642393	7	*EGFR*	Cell cycle	55245647	C	0.3	0.26	Dominant	1.07	0.70–1.63	0.754	
								Recessive	2.21	1.13–4.35	0.021	
								Additive	1.23	0.89–1.69	0.214	
								Allelic			0.227	1.46
rs12450550	17	*EME1*	DNA Repair	48456193	C	0.38	0.28	Dominant	1.28	0.84–1.95	0.257	
								Recessive	2.48	1.42–4.33	0.001	
								Additive	1.42	1.06–1.91	0.019	
								Allelic			0.004	8.39
rs170548	11	*ATM*	DNA Repair	108234836	C	0.36	0.31	Dominant	1.57	1.02–2.42	0.041	
								Recessive	0.98	0.49–1.92	0.947	
								Additive	1.27	0.93–1.72	0.135	
								Allelic			0.096	2.78
rs1035938	19	*GLTSCR1*	DNA Repair	48183771	T	0.29	0.24	Dominant	1.15	0.75–1.76	0.513	
								Recessive	2.14	1.09–4.19	0.027	
								Additive	1.27	0.92–1.74	0.145	
								Allelic			0.085	2.96
rs7721416	5	*XRCC4*	DNA Repair	82434993	A	0.41	0.47	Dominant	0.85	0.54–1.35	0.494	
								Recessive	0.51	0.27–0.94	0.032	
								Additive	0.76	0.56–1.04	0.089	
								Allelic			0.083	2.99
rs2662242	5	*XRCC4*	DNA Repair	82484885	C	0.42	0.48	Dominant	0.92	0.57–1.47	0.720	
								Recessive	0.49	0.26–0.91	0.024	
								Additive	0.78	0.57–1.06	0.114	
								Allelic			0.104	2.65

a MAF=Minor Allele Frequency

b OR adjusted for age, sex, and country.

Non-significant findings as well as the significant findings with wide confidence intervals are shown in the online appendix Tables S1–S3.

Strong LD (D' ≥ 0.95) was observed between four genotyped SNPs in *EGFR* (rs730437, rs11506105, rs4947986, and rs3752651) in which five haplotypes with frequency of > 1% were detected. In the *EGFR* block, the distribution of haplotypes was suggestively different between PBT patients and controls (χ^2^ = 8.1, df = 4, *p* = 0.089) and the most common haplotype (CGGT) had a significant risk effect compared with the other haplotypes combined (OR 1.31 [95% CI 1.05–1.64], *p* = 0.017) (Table 5). In the astrocytoma subgroup, the *CHAF1A* block (rs243341, rs105038, rs243356, and rs2992) with three haplotypes with frequency of >1% was found; however, the distribution of haplotypes in this haploblock was not significantly different between patients and controls (χ^2^ = 2.95, df = 2, *p* = 0.229). Moreover, in the non-astrocytoma subgroup, two haploblocks including *ATM* (rs228599, rs664143, rs170548, rs3092993, and rs3092992) and *XRCC4* (rs7721416 and rs2662242) with, respectively, five and three haplotypes with frequency of > 1% were observed; nevertheless, in none of these haploblocks, the distribution of haplotypes was significantly different between patients and controls (χ^2^ = 1.92, df = 4, *p* = 0.751 and χ^2^ = 2.98, df = 2, *p* = 0.226, respectively).

**Table 5:** Haplotype analysis of SNPs in *EGFR*

SNPs	Haplotype	Frequency	OR^a^	95% CI	*P*
EGFR: rs730437, rs11506105, rs4947986, rs3752651	AAAC	0.21	0.92	0.69–1.23	0.569
	AAAT	0.08	0.70	0.44–1.11	0.126
	AAGT	0.14	0.82	0.59–1.15	0.247
	AGGT	0.06	0.69	0.41–1.15	0.153
	CGGT	0.51	1.31	1.05–1.64	0.017

a Odds ratio for haplotype compared with all other haplotypes adjusted for age, sex, and country.

As described above, 248 testing procedures were performed. When the Bonferroni correction is applied, the reference *p* value is 0.0002 for an experiment-wide significance level of 0.05, and 0.0004 for a significance level of 0.10; none of the reported associations met these limits. As summarized in Table S4, no significant SNP-SNP interactions were observed (*p* < 0.001).

## DISCUSSION

This study suggests that SNPs involved in cell cycle and DNA repair pathways previously linked with brain tumors in adults are also associated with pediatric brain tumorigenesis. The results indicate that the A alleles of *EGFR* rs730437 and rs11506105 may decrease susceptibility to PBTs, whereas the A allele of *ERCC1* rs3212986 may increase the risk of these tumors. Moreover, the C alleles of *CHAF1A* rs243341 and rs2992 as well as the T allele of *XRCC1* rs25487 were associated with a decreased risk of astrocytoma subtype. In addition, an increased risk of non-astrocytoma tumor subtype associated with the C alleles of *EGFR* rs9642393, *EME1* rs12450550, and *ATM* rs170548, and the T allele of *GLTSCR1* rs1035938 as well as a decreased risk of this subtype associated with the A allele of *XRCC4* rs7721416 and the C allele of *XRCC4* rs2662242 were detected.

Genetic and epigenetic aberrations involved in cell cycle and DNA repair pathways have been proposed to play a role in PBT pathogenesis and progression [21–25]. Overexpression of the epidermal growth factor receptor (EGFR), which plays an important role in cell growth and development through the cell cycle pathway, is shown to be common and correlated with tumor grade in pediatric brain tumors [26, 27]. The function of genetic variants of *EGFR* is still unclear; however, intronic variations of *EGFR* found in this study may affect EGFR expression and play a role in pediatric brain tumorigenesis.

DNA repair is an important mechanism to maintain genomic stability and its functional failure may lead to carcinogenesis. Somatic variations of DNA repair genes have been identified in PBTs [28–30]; however, no study has investigated the role of DNA repair gene variations in PBT etiology. In this study, we examined the association between PBT risk and 37 SNPs in 17 DNA repair genes, shown to be associated with risk of adult brain tumors. Of these, 9 SNPs in 7 DNA repair genes (*ERCC1*, *CHAF1A*, *XRCC1*, *EME1*, *ATM*, *GLTSCR1*, and *XRCC4*) were also associated with susceptibility to PBTs. A few studies have previously indicated that DNA repair gene alterations may increase the predisposition to cancer formation in children [31, 32]. Moreover, these variations might predict the tumor drug sensitivity and the treatment outcome [33].

The *ERCC1* gene encodes excision repair cross-complementing group 1 which is involved in DNA nucleotide excision repair (NER) pathway [34]. The result of association between the A allele of *ERCC1* rs3212986 and increased risk of PBTs is in line with our finding in a meta-analysis of this polymorphism in adult brain tumors [3]. *XRCC1* which encodes an enzyme named X-ray cross-complementing group 1, plays an important role in base excision repair pathway (BER) [35]. Chromatin assembly factor 1, subunit A (CHAF1A) is involved in DNA mismatch repair (MMR) during the correction of DNA replication errors [36]. *XRCC4* encodes X-ray repair complementing defective repair in Chinese hamster cells 4 that functions in the repair of DNA double-strand breaks (DSBs) produced by ionizing radiation and restriction enzymes [37].

*EME1* encodes essential meiotic structure-specific endonuclease 1 which forms an endonuclease complex with methyl methanesulfonate-sensitive UV-sensitive 81 protein (MUS81) and plays an important role in DNA repair and maintaining of genome integrity [38]. *ATM* encodes a protein kinase which is a member of phosphatidyl inositol 3-kinase-like kinase (PIKK) family. ATM is a key regulator of cell cycle checkpoint signaling pathways which responds to DNA strand breaks by inducing cell-cycle arrest and hence facilitates DNA repair [39]. *GLTSCR1* stands for glioma tumor suppressor candidate region 1 and *GLTSCR1* rs1035938 polymorphism alters a CpG site within the 5′ CpG island of the gene. This germ-line alteration might affect the transcription of *GLTSCR1* and other candi-date genes in the region [40].

The present study was conducted based on the largest series of pediatric brain tumor cases to date with the purpose to investigate the association between genetic polymorphisms along with four main pathways hypothesized to be involved in brain tumorigenesis and PBT risk. Although investigating the effect of environmental risk factors was not the aim of our study, the fact that we did not take into consideration the possible gene-environment interactions is a limitation of this work. Moreover, due to shortage of large enough pediatric patient materials with access to DNA samples, we were not able to replicate our findings. Therefore, additional studies are necessary to validate these results, explore the mechanisms through which these genetic polymorphisms influence cancer susceptibility, and to investigate gene-environment interactions underling risk of PBTs.

Since this study describes the association between genetic variations and risk of pediatric brain tumors, it provides an important starting point for understanding the mechanisms behind the PBT etiology that in turn might lead to identifying clinically meaningful genetic risk and protective factors and eventually cancer prevention and treatment.

In conclusion, the present study indicates that while the minor alleles of polymorphisms in *ERCC1* may increase the risk of PBTs, the minor alleles of SNPs in *EGFR* are associated with decreased susceptibility to these tumors. Furthermore, polymorphisms in *CHAF1A* and *XRCC1* are associated with the risk of astrocytoma, whereas SNPs in *EME1*, *ATM*, *GLTSCR1*, and *XRCC4* are associated with susceptibility to non-astrocytoma subtypes. Therefore, genetic polymorphisms in cell cycle and DNA repair pathways associated with susceptibility to adult brain tumors seem also to be associated with PBT risk suggesting that pediatric and adult brain tumors might share similar etiological pathways.

## MATERIALS AND METHODS

### Study subjects and procedures

This population-based case-control study is based on the Cefalo study, a large international study of brain tumors in children and adolescents conducted in Sweden, Denmark, Norway, and Switzerland. Details of the study methods have been described previously [41]. Briefly, all children aged 7–19 years during the period between 1 April 2004 and 31 August 2008, diagnosed with a primary intracranial brain tumor defined according to the International Classification of Childhood Cancer, third edition (ICCC-3), group III [42], restricted to ICD-O-3 location C71, were considered as case subjects. Cases were subclassified according to the fourth edition of the World Health Organization (WHO) classification of tumors of the central nervous system [43]. Participants with neurofibromatosis or tuberous sclerosis were excluded from analyses. Medulloblastoma cases were excluded here since they will be included in a separate study. Controls were randomly selected from the population registers and matched to the cases by age, sex, and geographic region. A total of 352 (82%) cases and 646 (71%) controls participated in the interviews. The study was approved by the National data protection boards and ethical committees in all participating countries, and written informed consent was obtained from all participants and/or their parents.

Saliva collection and DNA extraction were performed using the Oragene self-collection kit (DNA Genotek, Ottawa, ON, Canada) and the DNA yield was quantitated using PicoGreen (Invitrogen, Carlsbad, CA, USA), according to the standard protocols. This study, in total, included saliva DNA of 245 cases and 489 controls. Distribution of diagnostic subtypes among cases were 134 astrocytoma, 19 ependymoma, 7 intracranial embryonal tumors, 20 other gliomas, 49 other specified intracranial neoplasms, and 16 with unspecified intracranial neoplasms (Table 1).

### Selection of candidate SNPs and genotyping

The PubMed database was searched (up to December 2012) using combinations of the following terms: ‘brain tumor’, ‘single nucleotide polymorphism’, ‘association’, ‘gene’, ‘risk’, ‘case control’, ‘susceptibility’, and ‘polymorphism’ to identify all the published peer-reviewed candidate gene-association studies of brain tumors. All the statistically significant SNPs reported by childhood brain tumor genetic association studies [11–13] as well as the SNPs reported by at least two candidate gene-association studies as being statistically associated with the risk of adult brain tumors [3–9, 44–49] were selected for genotyping.

Of 68 selected SNPs, 63 SNPs were satisfactorily genotyped (success rate ≥ 80%). Genotyping was performed at the Mutation Analysis core Facility (MAF), Clinical Research Centre, Huddinge University Hospital, Stockholm, Sweden, with staff blinded to sample status, using the Sequenom iPlex Gold platform with matrix-assisted laser desorption/ionization-time-of-flight (MALDI-TOF) mass spectrometry. The average success rate was 97% and the concordance rate for duplicate genotyping was 100%.

### Statistical analyses

The χ^2^ goodness-of-fit test was used to examine the consistency of allele frequencies with Hardy-Weinberg equilibrium (HWE) among the controls and *p* < 0.001 was considered statistically significant. Odds ratios (ORs) and corresponding 95% confidence intervals (CIs) were calculated using unconditional logistic regression modelling to evaluate the association between each SNP and the risk of PBT based on the Cochran–Armitage trend test of additivity (trend) as well as dominant (DOM) and recessive (REC) models, with adjustment for age, sex, and country. We applied the χ^2^ test to compare the allelic frequencies of the genotyped SNPs between cases and controls as well as the Wald test to evaluate the significance of interactions between SNPs and demographic variables including age, sex and country. SNP-SNP interactions were investigated for pairwise combinations of all the SNPs involved in the same pathway and *p* < 0.001 was set as the significance level of interaction analyses. Stratified analyses were performed by astrocytoma alone and the other tumor types combined (including ependymoma, intracranial embryonal tumors (except medulloblastoma), other gliomas, other specified intracranial neoplasms, and unspecified intracranial neoplasm); in order to increase the statistical power. To measure the linkage disequilibrium (LD) between the genotyped SNPs, D' was calculated. Haploblocks were defined based on the default LD block parameters in Haploview v4.2. Haplotype analyses were performed for the haplotype blocks harboring the SNPs that were found to be associated with PBTs. Haplotypes with a frequency > 1% were considered in the blocks with different haplotype distribution between cases and controls (*p* < 0.1). Although selection of SNPs for the analyses was based on a priori knowledge from candidate gene-association studies on adults, the possibility of false-positive findings was considered by providing the reference *p* value for an experiment-wide significance with Bonferroni correction (0.0002 for an experiment-wide significance level of 0.05, and 0.0004 for a significance level of 0.10). The analyses were carried out using PLINK v1.07 [50] and SAS statistical software version 9.3 (SAS Institute, Inc., Cary, NC, USA).

### URLs

PLINK: http://pngu.mgh.harvard.edu/~purcell/plink/

SAS: http://www.sas.com/

## SUPPLEMENTARY MATERIALS TABLES








