# Supplementary material for: Common genetic variations in cell cycle and DNA repair pathways associated with pediatric brain tumor susceptibility
Source: Oncotarget. 2016 Aug 24;7(39):63640–50. doi: 10.18632/oncotarget.11575 (PMC5325391; doi:10.18632/oncotarget.11575)
Supplement: Supplementary file 1 [file oncotarget-07-63640-s001.pdf]

Common genetic variations in cell cycle and DNA repair pathways associated with pediatric brain tumor susceptibility

Supplementary Materials

Supplementary Table S1: Summary results for SNPs unassociated with pediatric brain tumors.
See Supplementary_Table_S1

Supplementary Table S2: Summary results for SNPs unassociated with astrocytoma subtype.
See Supplementary_Table_S2

Supplementary Table S3: Summary results for SNPs unassociated with non-astrocytoma subtypes.
See Supplementary_Table_S3

Supplementary Table S4: Summary results of interaction analyses between pairwise SNPs involved in the same pathway

SNP 1			SNP2			OR	95% CI	*P*	Pathway
SNP	Chr.	Gene	SNP	Chr.	Gene				
rs1047840	1	*EXO1*	rs3777015	5	*XRCC4*	2.59	1.04–6.46	0.042	DNA Repair
rs1047840	1	*EXO1*	rs7003908	8	*PRKDC*	1.47	1.04–2.08	0.029	DNA Repair
rs1047840	1	*EXO1*	rs13181	19	*ERCC2*	1.55	1.09–2.19	0.014	DNA Repair
rs1047840	1	*EXO1*	rs3212986	19	*CD3EAP*	1.58	1.09–2.29	0.017	DNA Repair
rs828704	2	*XRCC5*	rs7715771	5	*XRCC4*	2.93	1.13–7.65	0.028	DNA Repair
rs828704	2	*XRCC5*	rs3092993	11	*ATM*	0.50	0.27–0.93	0.029	DNA Repair
rs828704	2	*XRCC5*	rs12450550	17	*EME1*	0.61	0.41–0.92	0.017	DNA Repair
rs828704	2	*XRCC5*	rs3212986	19	*CD3EAP*	1.71	1.07–2.72	0.024	DNA Repair
rs7721416	5	*XRCC5*	rs3093737	13	*LIG4*	1.76	1.02–3.02	0.042	DNA Repair
rs7721416	5	*XRCC5*	rs3212986	19	*CD3EAP*	1.64	1.10–2.44	0.015	DNA Repair
rs7721416	5	*XRCC5*	rs1035938	19	*GLTSCR1*	0.61	0.42–0.88	0.008	DNA Repair
rs2662242	5	*XRCC4*	rs1035938	19	*GLTSCR1*	0.58	0.40–0.83	0.003	DNA Repair
rs13161662	5	*XRCC4*	rs7715771	5	*XRCC4*	0.26	0.08–0.85	0.026	DNA Repair
rs13161662	5	*XRCC4*	rs2308321	10	*MGMT*	0.50	0.28–0.89	0.020	DNA Repair
rs13161662	5	*XRCC4*	rs1035938	19	*GLTSCR1*	1.68	1.09–2.57	0.017	DNA Repair
rs3777015	5	*XRCC4*	rs170548	11	*ATM*	2.54	1.07–5.99	0.034	DNA Repair
rs3777015	5	*XRCC4*	rs12450550	17	*EME1*	0.34	0.13–0.88	0.027	DNA Repair
rs2308321	10	*MGMT*	rs1035938	19	*GLTSCR1*	0.57	0.34–0.95	0.033	DNA Repair
rs664143	11	*ATM*	rs3092993	11	*ATM*	2.04	1.08–3.87	0.029	DNA Repair
rs3092993	11	*ATM*	rs238406	19	*ERCC2*	1.95	1.15–3.29	0.013	DNA Repair
rs861530	14	*XRCC3*	rs105038	19	*CHAF1A*	1.54	1.00–2.37	0.049	DNA Repair
rs861530	14	*XRCC3*	rs2992	19	*CHAF1A*	1.55	1.01–2.39	0.045	DNA Repair
rs243356	19	*CHAF1A*	rs13181	19	*ERCC2*	1.61	1.09–2.36	0.016	DNA Repair
rs238406	19	*ERCC2*	rs132771	22	*XRCC6*	1.68	1.01–2.79	0.046	DNA Repair
rs1801133	1	*MTHFR*	rs1805087	1	*MTR*	0.63	0.42–0.96	0.032	Metabolism
rs11579965	1	*NCF2*	rs1801275	16	*IL4R*	0.45	0.20–0.99	0.048	Inflammation
rs730437	7	*EGFR*	rs2682826	12	*EGFR*	0.69	0.49–0.97	0.033	Cell cycle
rs11506105	7	EGFR	rs2682826	12	*EGFR*	0.69	0.49–0.97	0.035	Cell cycle
