# Supplementary material for: Common genetic variations in cell cycle and DNA repair pathways associated with pediatric brain tumor susceptibility
Source: Oncotarget. 2016 Aug 24;7(39):63640–50. doi: 10.18632/oncotarget.11575 (PMC5325391; doi:10.18632/oncotarget.11575)
Supplement: Supplementary file 2 [file oncotarget-07-63640-s002.doc]

Table S1. Summary results for SNPs unassociated with pediatric brain tumors

| **SNP** | **Chr.** | **Location (bp)** | **Minor allele** | **MAF*^a^* in cases** | **MAF*^a^* in controls** | **Model** | **OR*^b^*** | **95% CI** | **P** | **CHISQ** |
| --- | --- | --- | --- | --- | --- | --- | --- | --- | --- | --- |
|  |  |  |  |  |  |  |  |  |  |  |
| rs1801131 | 1 | 11854476 | G | 0.31 | 0.32 | Dominant | 0.94 | 0.68-1.29 | 0.686 |  |
|  |  |  |  |  |  | Recessive | 0.86 | 0.50-1.47 | 0.578 |  |
|  |  |  |  |  |  | Additive | 0.93 | 0.73-1.19 | 0.575 |  |
|  |  |  |  |  |  | Allelic |  |  | 0.674 | 0.18 |
|  |  |  |  |  |  |  |  |  |  |  |
| rs1801133 | 1 | 11856378 | A | 0.32 | 0.28 | Dominant | 1.18 | 0.86-1.62 | 0.294 |  |
|  |  |  |  |  |  | Recessive | 1.41 | 0.84-2.36 | 0.189 |  |
|  |  |  |  |  |  | Additive | 1.18 | 0.93-1.50 | 0.165 |  |
|  |  |  |  |  |  | Allelic |  |  | 0.195 | 1.68 |
|  |  |  |  |  |  |  |  |  |  |  |
| rs3917727 | 1 | 169581258 | G | 0.32 | 0.36 | Dominant | 0.74 | 0.54-1.009 | 0.057 |  |
|  |  |  |  |  |  | Recessive | 1.04 | 0.66-1.663 | 0.856 |  |
|  |  |  |  |  |  | Additive | 0.86 | 0.68-1.083 | 0.200 |  |
|  |  |  |  |  |  | Allelic |  |  | 0.169 | 1.88 |
|  |  |  |  |  |  |  |  |  |  |  |
| rs11579965 | 1 | 183563161 | G | 0.07 | 0.08 | Dominant | 0.99 | 0.64-1.55 | 0.973 |  |
|  |  |  |  |  |  | Recessive | 0.68 | 0.07-6.60 | 0.737 |  |
|  |  |  |  |  |  | Additive | 0.98 | 0.64-1.49 | 0.921 |  |
|  |  |  |  |  |  | Allelic |  |  | 0.933 | 0.01 |
|  |  |  |  |  |  |  |  |  |  |  |
| rs1800896 | 1 | 206946897 | C | 0.46 | 0.47 | Dominant | 1.04 | 0.74-1.47 | 0.807 |  |
|  |  |  |  |  |  | Recessive | 0.89 | 0.61-1.29 | 0.523 |  |
|  |  |  |  |  |  | Additive | 0.98 | 0.79-1.21 | 0.826 |  |
|  |  |  |  |  |  | Allelic |  |  | 0.722 | 0.13 |
|  |  |  |  |  |  |  |  |  |  |  |
| rs1805087 | 1 | 237048500 | G | 0.19 | 0.18 | Dominant | 1.25 | 0.90-1.74 | 0.176 |  |
|  |  |  |  |  |  | Recessive | 0.93 | 0.41-2.12 | 0.860 |  |
|  |  |  |  |  |  | Additive | 1.17 | 0.88-1.54 | 0.279 |  |
|  |  |  |  |  |  | Allelic |  |  | 0.299 | 1.08 |
|  |  |  |  |  |  |  |  |  |  |  |
| rs1045485 | 2 | 202149589 | C | 0.14 | 0.12 | Dominant | 0.99 | 0.69-1.44 | 0.993 |  |
|  |  |  |  |  |  | Recessive | 4.84 | 1.46-16.04 | 0.01 |  |
|  |  |  |  |  |  | Additive | 1.14 | 0.82-1.57 | 0.436 |  |
|  |  |  |  |  |  | Allelic |  |  | 0.478 | 0.508 |
|  |  |  |  |  |  |  |  |  |  |  |
| rs1801394 | 5 | 7870973 | A | 0.45 | 0.46 | Dominant | 0.99 | 0.70-1.40 | 0.960 |  |
|  |  |  |  |  |  | Recessive | 0.88 | 0.59-1.31 | 0.524 |  |
|  |  |  |  |  |  | Additive | 0.95 | 0.76-1.19 | 0.687 |  |
|  |  |  |  |  |  | Allelic |  |  | 0.712 | 0.14 |
|  |  |  |  |  |  |  |  |  |  |  |
| rs1800925 | 5 | 131992809 | T | 0.22 | 0.20 | Dominant | 1.21 | 0.86-1.66 | 0.249 |  |
|  |  |  |  |  |  | Recessive | 0.75 | 0.32-1.73 | 0.494 |  |
|  |  |  |  |  |  | Additive | 1.11 | 0.84-1.46 | 0.456 |  |
|  |  |  |  |  |  | Allelic |  |  | 0.373 | 0.79 |
|  |  |  |  |  |  |  |  |  |  |  |
| rs20541 | 5 | 131995964 | A | 0.22 | 0.22 | Dominant | 1.05 | 0.76-1.44 | 0.776 |  |
|  |  |  |  |  |  | Recessive | 0.55 | 0.25-1.17 | 0.119 |  |
|  |  |  |  |  |  | Additive | 0.95 | 0.73-1.24 | 0.709 |  |
|  |  |  |  |  |  | Allelic |  |  | 0.767 | 0.09 |
|  |  |  |  |  |  |  |  |  |  |  |
| rs4947979 | 7 | 55195625 | G | 0.19 | 0.18 | Dominant | 1.00 | 0.72-1.39 | 0.996 |  |
|  |  |  |  |  |  | Recessive | 1.68 | 0.72-3.93 | 0.228 |  |
|  |  |  |  |  |  | Additive | 1.06 | 0.79-1.41 | 0.694 |  |
|  |  |  |  |  |  | Allelic |  |  | 0.726 | 0.12 |
|  |  |  |  |  |  |  |  |  |  |  |
| rs4947986 | 7 | 55221655 | A | 0.27 | 0.29 | Dominant | 0.86 | 0.63-1.18 | 0.355 |  |
|  |  |  |  |  |  | Recessive | 0.75 | 0.42-1.35 | 0.332 |  |
|  |  |  |  |  |  | Additive | 0.87 | 0.68-1.11 | 0.256 |  |
|  |  |  |  |  |  | Allelic |  |  | 0.275 | 1.19 |
|  |  |  |  |  |  |  |  |  |  |  |
| rs3752651 | 7 | 55229543 | C | 0.21 | 0.21 | Dominant | 0.96 | 0.69-1.34 | 0.805 |  |
|  |  |  |  |  |  | Recessive | 1.09 | 0.53-2.24 | 0.820 |  |
|  |  |  |  |  |  | Additive | 0.98 | 0.75-1.29 | 0.908 |  |
|  |  |  |  |  |  | Allelic |  |  | 0.905 | 0.01 |
|  |  |  |  |  |  |  |  |  |  |  |
| rs1468727 | 7 | 55230105 | T | 0.24 | 0.22 | Dominant | 1.04 | 0.76-1.43 | 0.809 |  |
|  |  |  |  |  |  | Recessive | 1.56 | 0.82-2.95 | 0.174 |  |
|  |  |  |  |  |  | Additive | 1.1 | 0.85-1.42 | 0.467 |  |
|  |  |  |  |  |  | Allelic |  |  | 0.486 | 0.49 |
|  |  |  |  |  |  |  |  |  |  |  |
| rs9642393 | 7 | 55245647 | C | 0.26 | 0.26 | Dominant | 0.89 | 0.65-1.23 | 0.484 |  |
|  |  |  |  |  |  | Recessive | 1.44 | 0.82-2.54 | 0.205 |  |
|  |  |  |  |  |  | Additive | 0.99 | 0.78-1.28 | 0.989 |  |
|  |  |  |  |  |  | Allelic |  |  | 0.979 | 0.00 |
|  |  |  |  |  |  |  |  |  |  |  |
| rs2291427 | 10 | 45936224 | A | 0.32 | 0.31 | Dominant | 1.08 | 0.79-1.48 | 0.624 |  |
|  |  |  |  |  |  | Recessive | 1.18 | 0.71-1.96 | 0.522 |  |
|  |  |  |  |  |  | Additive | 1.08 | 0.86-1.37 | 0.509 |  |
|  |  |  |  |  |  | Allelic |  |  | 0.539 | 0.38 |
|  |  |  |  |  |  |  |  |  |  |  |
| rs2031920 | 10 | 135339845 | T | 0.01 | 0.03 | Dominant | 0.44 | 0.16-1.18 | 0.101 |  |
|  |  |  |  |  |  | Recessive | NA | NA | NA |  |
|  |  |  |  |  |  | Additive | 0.44 | 0.16-1.18 | 0.101 |  |
|  |  |  |  |  |  | Allelic |  |  | 0.076 | 3.15 |
|  |  |  |  |  |  |  |  |  |  |  |
| rs1695 | 11 | 67352689 | G | 0.32 | 0.33 | Dominant | 1.05 | 0.77-1.44 | 0.753 |  |
|  |  |  |  |  |  | Recessive | 0.78 | 0.46-1.32 | 0.353 |  |
|  |  |  |  |  |  | Additive | 0.98 | 0.77-1.24 | 0.848 |  |
|  |  |  |  |  |  | Allelic |  |  | 0.763 | 0.09 |
|  |  |  |  |  |  |  |  |  |  |  |
| rs2682826 | 12 | 117652838 | A | 0.28 | 0.27 | Dominant | 1.18 | 0.86-1.62 | 0.3 |  |
|  |  |  |  |  |  | Recessive | 0.88 | 0.49-1.57 | 0.667 |  |
|  |  |  |  |  |  | Additive | 1.08 | 0.85-1.38 | 0.535 |  |
|  |  |  |  |  |  | Allelic |  |  | 0.559 | 0.34 |
|  |  |  |  |  |  |  |  |  |  |  |
| rs2606345 | 15 | 75017176 | C | 0.33 | 0.36 | Dominant | 0.83 | 0.60-1.13 | 0.230 |  |
|  |  |  |  |  |  | Recessive | 0.92 | 0.58-1.44 | 0.705 |  |
|  |  |  |  |  |  | Additive | 0.89 | 0.71-1.11 | 0.299 |  |
|  |  |  |  |  |  | Allelic |  |  | 0.261 | 1.26 |
|  |  |  |  |  |  |  |  |  |  |  |
| rs1801275 | 16 | 27374400 | G | 0.19 | 0.21 | Dominant | 0.99 | 0.72-1.38 | 0.989 |  |
|  |  |  |  |  |  | Recessive | 0.54 | 0.24-1.21 | 0.133 |  |
|  |  |  |  |  |  | Additive | 0.92 | 0.71-1.21 | 0.565 |  |
|  |  |  |  |  |  | Allelic |  |  | 0.636 | 0.22 |
|  |  |  |  |  |  |  |  |  |  |  |
| rs9303277 | 17 | 37976469 | C | 0.50 | 0.48 | Dominant | 1.04 | 0.73-1.49 | 0.819 |  |
|  |  |  |  |  |  | Recessive | 1.23 | 0.85-1.78 | 0.270 |  |
|  |  |  |  |  |  | Additive | 1.09 | 0.88-1.38 | 0.422 |  |
|  |  |  |  |  |  | Allelic |  |  | 0.379 | 0.77 |
|  |  |  |  |  |  |  |  |  |  |  |
| rs11557467 | 17 | 38028634 | G | 0.50 | 0.49 | Dominant | 0.99 | 0.69-1.43 | 0.982 |  |
|  |  |  |  |  |  | Recessive | 1.13 | 0.78-1.62 | 0.515 |  |
|  |  |  |  |  |  | Additive | 1.04 | 0.84-1.30 | 0.704 |  |
|  |  |  |  |  |  | Allelic |  |  | 0.647 | 0.21 |
|  |  |  |  |  |  |  |  |  |  |  |
| rs8067378 | 17 | 38051348 | A | 0.50 | 0.48 | Dominant | 0.98 | 0.68-1.40 | 0.902 |  |
|  |  |  |  |  |  | Recessive | 1.26 | 0.87-1.82 | 0.216 |  |
|  |  |  |  |  |  | Additive | 1.08 | 0.86-1.35 | 0.503 |  |
|  |  |  |  |  |  | Allelic |  |  | 0.452 | 0.57 |
|  |  |  |  |  |  |  |  |  |  |  |
| rs2290400 | 17 | 38066240 | T | 0.50 | 0.49 | Dominant | 0.98 | 0.69-1.40 | 0.914 |  |
|  |  |  |  |  |  | Recessive | 1.18 | 0.82-1.69 | 0.363 |  |
|  |  |  |  |  |  | Additive | 1.06 | 0.85-1.32 | 0.627 |  |
|  |  |  |  |  |  | Allelic |  |  | 0.576 | 0.31 |
|  |  |  |  |  |  |  |  |  |  |  |
| rs7216389 | 17 | 38069949 | T | 0.49 | 0.48 | Dominant | 0.96 | 0.67-1.38 | 0.845 |  |
|  |  |  |  |  |  | Recessive | 1.14 | 0.79-1.64 | 0.499 |  |
|  |  |  |  |  |  | Additive | 1.03 | 0.83-1.29 | 0.776 |  |
|  |  |  |  |  |  | Allelic |  |  | 0.678 | 0.17 |
|  |  |  |  |  |  |  |  |  |  |  |
| rs1136410 | 1 | 226555302 | G | 0.16 | 0.19 | Dominant | 0.85 | 0.60-1.19 | 0.347 |  |
|  |  |  |  |  |  | Recessive | 0.66 | 0.29-1.51 | 0.321 |  |
|  |  |  |  |  |  | Additive | 0.85 | 0.64-1.13 | 0.256 |  |
|  |  |  |  |  |  | Allelic |  |  | 0.221 | 1.49 |
|  |  |  |  |  |  |  |  |  |  |  |
| rs1047840 | 1 | 242042301 | A | 0.36 | 0.38 | Dominant | 0.85 | 0.62-1.16 | 0.304 |  |
|  |  |  |  |  |  | Recessive | 1.01 | 0.65-1.57 | 0.964 |  |
|  |  |  |  |  |  | Additive | 0.92 | 0.74-1.16 | 0.485 |  |
|  |  |  |  |  |  | Allelic |  |  | 0.527 | 0.40 |
|  |  |  |  |  |  |  |  |  |  |  |
| rs828704 | 2 | 216993611 | C | 0.22 | 0.19 | Dominant | 1.16 | 0.84-1.60 | 0.373 |  |
|  |  |  |  |  |  | Recessive | 1.24 | 0.61-2.51 | 0.546 |  |
|  |  |  |  |  |  | Additive | 1.14 | 0.87-1.48 | 0.338 |  |
|  |  |  |  |  |  | Allelic |  |  | 0.249 | 1.33 |
|  |  |  |  |  |  |  |  |  |  |  |
| rs7721416 | 5 | 82434993 | A | 0.42 | 0.47 | Dominant | 0.81 | 0.57-1.13 | 0.215 |  |
|  |  |  |  |  |  | Recessive | 0.75 | 0.49-1.13 | 0.165 |  |
|  |  |  |  |  |  | Additive | 0.83 | 0.66-1.04 | 0.106 |  |
|  |  |  |  |  |  | Allelic |  |  | 0.101 | 2.69 |
|  |  |  |  |  |  |  |  |  |  |  |
| rs2662242 | 5 | 82484885 | C | 0.44 | 0.48 | Dominant | 0.81 | 0.57-1.15 | 0.234 |  |
|  |  |  |  |  |  | Recessive | 0.79 | 0.53-1.18 | 0.255 |  |
|  |  |  |  |  |  | Additive | 0.85 | 0.67-1.06 | 0.151 |  |
|  |  |  |  |  |  | Allelic |  |  | 0.138 | 2.20 |
|  |  |  |  |  |  |  |  |  |  |  |
| rs16900208 | 5 | 82489315 | G | 0 | 0.00 | Dominant | 9.092e-010 | 0-inf | 0.999 |  |
|  |  |  |  |  |  | Recessive | NA | NA | NA |  |
|  |  |  |  |  |  | Additive | 9.092e-010 | 0-inf | 0.999 |  |
|  |  |  |  |  |  | Allelic |  |  | 0.476 | 0.51 |
|  |  |  |  |  |  |  |  |  |  |  |
| rs13161662 | 5 | 82505596 | G | 0.41 | 0.39 | Dominant | 1.18 | 0.81-1.72 | 0.398 |  |
|  |  |  |  |  |  | Recessive | 1.08 | 0.66-1.78 | 0.758 |  |
|  |  |  |  |  |  | Additive | 1.11 | 0.86-1.44 | 0.452 |  |
|  |  |  |  |  |  | Allelic |  |  | 0.442 | 0.59 |
|  |  |  |  |  |  |  |  |  |  |  |
| rs7715771 | 5 | 82521868 | T | 0.06 | 0.04 | Dominant | 1.42 | 0.84-2.39 | 0.192 |  |
|  |  |  |  |  |  | Recessive | 1.46 | 0.09-23.68 | 0.791 |  |
|  |  |  |  |  |  | Additive | 1.39 | 0.84-2.29 | 0.198 |  |
|  |  |  |  |  |  | Allelic |  |  | 0.209 | 1.58 |
|  |  |  |  |  |  |  |  |  |  |  |
| rs3777015 | 5 | 82648883 | G | 0.05 | 0.04 | Dominant | 1.24 | 0.69-2.24 | 0.473 |  |
|  |  |  |  |  |  | Recessive | NA | NA | NA |  |
|  |  |  |  |  |  | Additive | 1.24 | 0.69-2.24 | 0.473 |  |
|  |  |  |  |  |  | Allelic |  |  | 0.391 | 0.74 |
|  |  |  |  |  |  |  |  |  |  |  |
| rs1805377 | 5 | 82648943 | A | 0.13 | 0.13 | Dominant | 1.03 | 0.71-1.49 | 0.874 |  |
|  |  |  |  |  |  | Recessive | 0.46 | 0.13-1.65 | 0.232 |  |
|  |  |  |  |  |  | Additive | 0.96 | 0.69-1.34 | 0.821 |  |
|  |  |  |  |  |  | Allelic |  |  | 0.943 | 0.01 |
|  |  |  |  |  |  |  |  |  |  |  |
| rs1056503 | 5 | 82648977 | G | 0.13 | 0.13 | Dominant | 1.09 | 0.76-1.57 | 0.636 |  |
|  |  |  |  |  |  | Recessive | 0.45 | 0.13-1.64 | 0.227 |  |
|  |  |  |  |  |  | Additive | 1.01 | 0.73-1.39 | 0.963 |  |
|  |  |  |  |  |  | Allelic |  |  | 0.848 | 0.04 |
|  |  |  |  |  |  |  |  |  |  |  |
| rs7003908 | 8 | 48770702 | C | 0.33 | 0.35 | Dominant | 0.88 | 0.64-1.21 | 0.422 |  |
|  |  |  |  |  |  | Recessive | 0.81 | 0.49-1.36 | 0.429 |  |
|  |  |  |  |  |  | Additive | 0.89 | 0.69-1.13 | 0.331 |  |
|  |  |  |  |  |  | Allelic |  |  | 0.363 | 0.83 |
|  |  |  |  |  |  |  |  |  |  |  |
| rs12917 | 10 | 131506283 | T | 0.14 | 0.12 | Dominant | 1.36 | 0.95-1.95 | 0.094 |  |
|  |  |  |  |  |  | Recessive | 0.84 | 0.21-3.29 | 0.799 |  |
|  |  |  |  |  |  | Additive | 1.28 | 0.92-1.78 | 0.142 |  |
|  |  |  |  |  |  | Allelic |  |  | 0.179 | 1.81 |
|  |  |  |  |  |  |  |  |  |  |  |
| rs2308321 | 10 | 131565064 | G | 0.13 | 0.13 | Dominant | 1.03 | 0.72-1.48 | 0.870 |  |
|  |  |  |  |  |  | Recessive | 0.24 | 0.03-1.89 | 0.175 |  |
|  |  |  |  |  |  | Additive | 0.96 | 0.69-1.35 | 0.832 |  |
|  |  |  |  |  |  | Allelic |  |  | 0.748 | 0.10 |
|  |  |  |  |  |  |  |  |  |  |  |
| rs228599 | 11 | 108107660 | G | 0.44 | 0.45 | Dominant | 1.14 | 0.81-1.60 | 0.462 |  |
|  |  |  |  |  |  | Recessive | 0.78 | 0.52-1.16 | 0.225 |  |
|  |  |  |  |  |  | Additive | 0.98 | 0.78-1.22 | 0.832 |  |
|  |  |  |  |  |  | Allelic |  |  | 0.893 | 0.02 |
|  |  |  |  |  |  |  |  |  |  |  |
| rs3092992 | 11 | 108195779 | C | 0.05 | 0.05 | Dominant | 0.99 | 0.58-1.72 | 0.998 |  |
|  |  |  |  |  |  | Recessive | 1.09e-009 | 0-inf | 0.999 |  |
|  |  |  |  |  |  | Additive | 0.95 | 0.56-1.6 | 0.848 |  |
|  |  |  |  |  |  | Allelic |  |  | 0.861 | 0.03 |
|  |  |  |  |  |  |  |  |  |  |  |
| rs664143 | 11 | 108225661 | A | 0.44 | 0.45 | Dominant | 1.13 | 0.81-1.59 | 0.471 |  |
|  |  |  |  |  |  | Recessive | 0.7 | 0.51-1.14 | 0.185 |  |
|  |  |  |  |  |  | Additive | 0.97 | 0.78-1.21 | 0.777 |  |
|  |  |  |  |  |  | Allelic |  |  | 0.813 | 0.06 |
|  |  |  |  |  |  |  |  |  |  |  |
| rs170548 | 11 | 108234836 | C | 0.34 | 0.31 | Dominant | 1.24 | 0.90-1.69 | 0.189 |  |
|  |  |  |  |  |  | Recessive | 1.08 | 0.66-1.77 | 0.754 |  |
|  |  |  |  |  |  | Additive | 1.14 | 0.91-1.44 | 0.263 |  |
|  |  |  |  |  |  | Allelic |  |  | 0.222 | 1.49 |
|  |  |  |  |  |  |  |  |  |  |  |
| rs3092993 | 11 | 108235115 | A | 0.12 | 0.14 | Dominant | 0.82 | 0.57-1.18 | 0.293 |  |
|  |  |  |  |  |  | Recessive | 1.05 | 0.26-4.29 | 0.944 |  |
|  |  |  |  |  |  | Additive | 0.85 | 0.60-1.19 | 0.336 |  |
|  |  |  |  |  |  | Allelic |  |  | 0.287 | 1.14 |
|  |  |  |  |  |  |  |  |  |  |  |
| rs3093739 | 13 | 108867401 | G | 0.13 | 0.14 | Dominant | 0.92 | 0.64-1.33 | 0.666 |  |
|  |  |  |  |  |  | Recessive | 1.04 | 0.31-3.55 | 0.944 |  |
|  |  |  |  |  |  | Additive | 0.94 | 0.68-1.31 | 0.709 |  |
|  |  |  |  |  |  | Allelic |  |  | 0.631 | 0.23 |
|  |  |  |  |  |  |  |  |  |  |  |
| rs3093737 | 13 | 108867483 | C | 0.09 | 0.09 | Dominant | 0.98 | 0.64-1.49 | 0.913 |  |
|  |  |  |  |  |  | Recessive | 1.63 | 0.36-7.46 | 0.530 |  |
|  |  |  |  |  |  | Additive | 1.01 | 0.69-1.49 | 0.959 |  |
|  |  |  |  |  |  | Allelic |  |  | 0.929 | 0.01 |
|  |  |  |  |  |  |  |  |  |  |  |
| rs3212092 | 14 | 104168644 | A | 0.01 | 0.01 | Dominant | 2.49 | 0.82-7.56 | 0.108 |  |
|  |  |  |  |  |  | Recessive | NA | NA | NA |  |
|  |  |  |  |  |  | Additive | 2.49 | 0.82-7.58 | 0.108 |  |
|  |  |  |  |  |  | Allelic |  |  | 0.117 | 2.46 |
|  |  |  |  |  |  |  |  |  |  |  |
| rs861530 | 14 | 104174123 | T | 0.26 | 0.30 | Dominant | 0.72 | 0.50-1.03 | 0.071 |  |
|  |  |  |  |  |  | Recessive | 0.87 | 0.49-1.55 | 0.643 |  |
|  |  |  |  |  |  | Additive | 0.81 | 0.62-1.06 | 0.125 |  |
|  |  |  |  |  |  | Allelic |  |  | 0.102 | 2.68 |
|  |  |  |  |  |  |  |  |  |  |  |
| rs1625895 | 17 | 7578115 | T | 0.13 | 0.13 | Dominant | 0.99 | 0.69-1.41 | 0.936 |  |
|  |  |  |  |  |  | Recessive | 1.09 | 0.31-3.78 | 0.894 |  |
|  |  |  |  |  |  | Additive | 0.99 | 0.72-1.38 | 0.969 |  |
|  |  |  |  |  |  | Allelic |  |  | 0.974 | 0.00 |
|  |  |  |  |  |  |  |  |  |  |  |
| rs2287499 | 17 | 7592168 | G | 0.13 | 0.14 | Dominant | 0.90 | 0.63-1.31 | 0.591 |  |
|  |  |  |  |  |  | Recessive | 0.63 | 0.19-2.04 | 0.445 |  |
|  |  |  |  |  |  | Additive | 0.89 | 0.64-1.23 | 0.483 |  |
|  |  |  |  |  |  | Allelic |  |  | 0.619 | 0.25 |
|  |  |  |  |  |  |  |  |  |  |  |
| rs12450550 | 17 | 48456193 | C | 0.33 | 0.28 | Dominant | 1.20 | 0.88-1.65 | 0.244 |  |
|  |  |  |  |  |  | Recessive | 1.33 | 0.81-2.18 | 0.269 |  |
|  |  |  |  |  |  | Additive | 1.18 | 0.93-1.48 | 0.169 |  |
|  |  |  |  |  |  | Allelic |  |  | 0.104 | 2.64 |
|  |  |  |  |  |  |  |  |  |  |  |
| rs243341 | 19 | 4405106 | C | 0.26 | 0.29 | Dominant | 0.84 | 0.61-1.14 | 0.262 |  |
|  |  |  |  |  |  | Recessive | 0.59 | 0.31-1.09 | 0.093 |  |
|  |  |  |  |  |  | Additive | 0.82 | 0.64-1.05 | 0.109 |  |
|  |  |  |  |  |  | Allelic |  |  | 0.121 | 2.4 |
|  |  |  |  |  |  |  |  |  |  |  |
| rs105038 | 19 | 4414710 | T | 0.26 | 0.29 | Dominant | 0.88 | 0.64-1.19 | 0.406 |  |
|  |  |  |  |  |  | Recessive | 0.64 | 0.35-1.17 | 0.149 |  |
|  |  |  |  |  |  | Additive | 0.85 | 0.67-1.09 | 0.204 |  |
|  |  |  |  |  |  | Allelic |  |  | 0.217 | 1.52 |
|  |  |  |  |  |  |  |  |  |  |  |
| rs243356 | 19 | 4415452 | T | 0.23 | 0.24 | Dominant | 0.95 | 0.69-1.31 | 0.758 |  |
|  |  |  |  |  |  | Recessive | 0.66 | 0.34-1.26 | 0.208 |  |
|  |  |  |  |  |  | Additive | 0.91 | 0.70-1.17 | 0.446 |  |
|  |  |  |  |  |  | Allelic |  |  | 0.459 | 0.55 |
|  |  |  |  |  |  |  |  |  |  |  |
| rs2992 | 19 | 4443046 | C | 0.26 | 0.29 | Dominant | 0.86 | 0.63-1.17 | 0.339 |  |
|  |  |  |  |  |  | Recessive | 0.59 | 0.32-1.12 | 0.109 |  |
|  |  |  |  |  |  | Additive | 0.83 | 0.65-1.07 | 0.151 |  |
|  |  |  |  |  |  | Allelic |  |  | 0.159 | 1.99 |
|  |  |  |  |  |  |  |  |  |  |  |
| rs25487 | 19 | 44055726 | T | 0.32 | 0.36 | Dominant | 0.76 | 0.56-1.04 | 0.089 |  |
|  |  |  |  |  |  | Recessive | 0.85 | 0.52-1.38 | 0.517 |  |
|  |  |  |  |  |  | Additive | 0.83 | 0.66-1.05 | 0.119 |  |
|  |  |  |  |  |  | Allelic |  |  | 0.119 | 2.43 |
|  |  |  |  |  |  |  |  |  |  |  |
| rs13181 | 19 | 45854919 | G | 0.37 | 0.36 | Dominant | 1.16 | 0.84-1.59 | 0.369 |  |
|  |  |  |  |  |  | Recessive | 0.88 | 0.54-1.44 | 0.616 |  |
|  |  |  |  |  |  | Additive | 1.05 | 0.831.33 | 0.677 |  |
|  |  |  |  |  |  | Allelic |  |  | 0.626 | 0.24 |
|  |  |  |  |  |  |  |  |  |  |  |
| rs238406 | 19 | 45868309 | T | 0.45 | 0.45 | Dominant | 1.02 | 0.72-1.45 | 0.898 |  |
|  |  |  |  |  |  | Recessive | 1.08 | 0.72-1.62 | 0.702 |  |
|  |  |  |  |  |  | Additive | 1.04 | 0.82-1.31 | 0.761 |  |
|  |  |  |  |  |  | Allelic |  |  | 0.835 | 0.04 |
|  |  |  |  |  |  |  |  |  |  |  |
| rs1035938 | 19 | 48183771 | T | 0.28 | 0.24 | Dominant | 1.22 | 0.89-1.67 | 0.217 |  |
|  |  |  |  |  |  | Recessive | 1.39 | 0.78-2.49 | 0.261 |  |
|  |  |  |  |  |  | Additive | 1.198 | 0.94-1.53 | 0.150 |  |
|  |  |  |  |  |  | Allelic |  |  | 0.097 | 2.75 |
|  |  |  |  |  |  |  |  |  |  |  |
| rs20579 | 19 | 48668830 | A | 0.11 | 0.13 | Dominant | 0.86 | 0.59-1.25 | 0.435 |  |
|  |  |  |  |  |  | Recessive | 0.37 | 0.043-3.22 | 0.368 |  |
|  |  |  |  |  |  | Additive | 0.85 | 0.59-1.21 | 0.353 |  |
|  |  |  |  |  |  | Allelic |  |  | 0.350 | 0.87 |
|  |  |  |  |  |  |  |  |  |  |  |
| rs132771 | 22 | 42025350 | A | 0.15 | 0.16 | Dominant | 0.95 | 0.67-1.34 | 0.767 |  |
|  |  |  |  |  |  | Recessive | 0.19 | 0.02-1.51 | 0.116 |  |
|  |  |  |  |  |  | Additive | 0.89 | 0.65-1.23 | 0.477 |  |
|  |  |  |  |  |  | Allelic |  |  | 0.539 | 0.38 |

***a***: MAF=Minor Allele Frequency ***b*:** OR adjusted for age, sex, and country
