# Supplementary material for: Common genetic variations in cell cycle and DNA repair pathways associated with pediatric brain tumor susceptibility
Source: Oncotarget. 2016 Aug 24;7(39):63640–50. doi: 10.18632/oncotarget.11575 (PMC5325391; doi:10.18632/oncotarget.11575)
Supplement: Supplementary file 3 [file oncotarget-07-63640-s003.doc]

**Table S2. Summary results for SNPs unassociated with astrocytoma subtype**

| **SNP** | **Chr.** | **Location (bp)** | **Minor allele** | **MAF*^a^* in cases** | **MAF*^a^* in controls** | **Model** | **OR*^b^*** | **95% CI** | **P** | **CHISQ** |
| --- | --- | --- | --- | --- | --- | --- | --- | --- | --- | --- |
|  |  |  |  |  |  |  |  |  |  |  |
| rs1801131 | 1 | 11854476 | G | 0.31 | 0.32 | Dominant | 0.96 | 0.64-1.42 | 0.828 |  |
|  |  |  |  |  |  | Recessive | 0.82 | 0.41-1.64 | 0.577 |  |
|  |  |  |  |  |  | Additive | 0.94 | 0.69-1.27 | 0.675 |  |
|  |  |  |  |  |  | Allelic |  |  | 0.689 | 0.16 |
|  |  |  |  |  |  |  |  |  |  |  |
| rs1801133 | 1 | 11856378 | A | 0.31 | 0.28 | Dominant | 1.04 | 0.69-1.54 | 0.863 |  |
|  |  |  |  |  |  | Recessive | 1.44 | 0.78-2.67 | 0.246 |  |
|  |  |  |  |  |  | Additive | 1.10 | 0.82-1.48 | 0.512 |  |
|  |  |  |  |  |  | Allelic |  |  | 0.457 | 0.55 |
|  |  |  |  |  |  |  |  |  |  |  |
| rs3917727 | 1 | 169581258 | G | 0.33 | 0.35 | Dominant | 0.80 | 0.54-1.19 | 0.267 |  |
|  |  |  |  |  |  | Recessive | 1.02 | 0.57-1.82 | 0.957 |  |
|  |  |  |  |  |  | Additive | 0.89 | 0.67-1.19 | 0.437 |  |
|  |  |  |  |  |  | Allelic |  |  | 0.420 | 0.65 |
|  |  |  |  |  |  |  |  |  |  |  |
| rs11579965 | 1 | 183563161 | G | 0.09 | 0.08 | Dominant | 1.18 | 0.69-1.99 | 0.542 |  |
|  |  |  |  |  |  | Recessive | 1.20 | 0.12-11.79 | 0.875 |  |
|  |  |  |  |  |  | Additive | 1.16 | 0.71-1.89 | 0.545 |  |
|  |  |  |  |  |  | Allelic |  |  | 0.526 | 0.40 |
|  |  |  |  |  |  |  |  |  |  |  |
| rs1800896 | 1 | 206946897 | C | 0.49 | 0.47 | Dominant | 1.14 | 0.74-1.76 | 0.556 |  |
|  |  |  |  |  |  | Recessive | 1.09 | 0.69-1.69 | 0.718 |  |
|  |  |  |  |  |  | Additive | 1.08 | 0.83-1.41 | 0.563 |  |
|  |  |  |  |  |  | Allelic |  |  | 0.573 | 0.32 |
|  |  |  |  |  |  |  |  |  |  |  |
| rs1805087 | 1 | 237048500 | G | 0.20 | 0.18 | Dominant | 1.37 | 0.92-2.05 | 0.123 |  |
|  |  |  |  |  |  | Recessive | 0.59 | 0.17-2.06 | 0.413 |  |
|  |  |  |  |  |  | Additive | 1.20 | 0.85-1.69 | 0.294 |  |
|  |  |  |  |  |  | Allelic |  |  | 0.308 | 1.04 |
|  |  |  |  |  |  |  |  |  |  |  |
| rs1045485 | 2 | 202149589 | C | 0.15 | 0.12 | Dominant | 1.08 | 0.69-1.69 | 0.750 |  |
|  |  |  |  |  |  | Recessive | 5.79 | 1.59-21.14 | 0.008 |  |
|  |  |  |  |  |  | Additive | 1.24 | 0.84-1.84 | 0.278 |  |
|  |  |  |  |  |  | Allelic |  |  | 0.291 | 1.11 |
|  |  |  |  |  |  |  |  |  |  |  |
| rs1801394 | 5 | 7870973 | A | 0.47 | 0.46 | Dominant | 1.06 | 0.69-1.64 | 0.792 |  |
|  |  |  |  |  |  | Recessive | 1.08 | 0.67-1.73 | 0.752 |  |
|  |  |  |  |  |  | Additive | 1.05 | 0.79-1.39 | 0.723 |  |
|  |  |  |  |  |  | Allelic |  |  | 0.699 | 0.15 |
|  |  |  |  |  |  |  |  |  |  |  |
| rs1800925 | 5 | 131992809 | T | 0.22 | 0.20 | Dominant | 1.13 | 0.76-1.68 | 0.556 |  |
|  |  |  |  |  |  | Recessive | 1.07 | 0.42-2.72 | 0.891 |  |
|  |  |  |  |  |  | Additive | 1.09 | 0.79-1.53 | 0.586 |  |
|  |  |  |  |  |  | Allelic |  |  | 0.568 | 0.33 |
|  |  |  |  |  |  |  |  |  |  |  |
| rs20541 | 5 | 131995964 | A | 0.22 | 0.22 | Dominant | 1.13 | 0.77-1.68 | 0.529 |  |
|  |  |  |  |  |  | Recessive | 0.33 | 0.10-1.11 | 0.073 |  |
|  |  |  |  |  |  | Additive | 0.97 | 0.69-1.34 | 0.837 |  |
|  |  |  |  |  |  | Allelic |  |  | 0.835 | 0.04 |
|  |  |  |  |  |  |  |  |  |  |  |
| rs4947979 | 7 | 55195625 | G | 0.17 | 0.18 | Dominant | 0.82 | 0.54-1.25 | 0.358 |  |
|  |  |  |  |  |  | Recessive | 2.09 | 0.81-5.39 | 0.126 |  |
|  |  |  |  |  |  | Additive | 0.95 | 0.66-1.36 | 0.775 |  |
|  |  |  |  |  |  | Allelic |  |  | 0.759 | 0.09 |
|  |  |  |  |  |  |  |  |  |  |  |
| rs11506105 | 7 | 55220177 | A | 0.39 | 0.45 | Dominant | 0.71 | 0.47-1.06 | 0.089 |  |
|  |  |  |  |  |  | Recessive | 0.89 | 0.55-1.44 | 0.629 |  |
|  |  |  |  |  |  | Additive | 0.83 | 0.63-1.08 | 0.171 |  |
|  |  |  |  |  |  | Allelic |  |  | 0.175 | 1.84 |
|  |  |  |  |  |  |  |  |  |  |  |
| rs4947986 | 7 | 55221655 | A | 0.28 | 0.29 | Dominant | 0.86 | 0.57-1.27 | 0.441 |  |
|  |  |  |  |  |  | Recessive | 1.01 | 0.51-1.98 | 0.977 |  |
|  |  |  |  |  |  | Additive | 0.92 | 0.67-1.24 | 0.568 |  |
|  |  |  |  |  |  | Allelic |  |  | 0.576 | 0.31 |
|  |  |  |  |  |  |  |  |  |  |  |
| rs3752651 | 7 | 55229543 | C | 0.23 | 0.21 | Dominant | 1.05 | 0.69-1.59 | 0.838 |  |
|  |  |  |  |  |  | Recessive | 1.19 | 0.49-2.85 | 0.695 |  |
|  |  |  |  |  |  | Additive | 1.06 | 0.75-1.49 | 0.753 |  |
|  |  |  |  |  |  | Allelic |  |  | 0.758 | 0.09 |
|  |  |  |  |  |  |  |  |  |  |  |
| rs1468727 | 7 | 55230105 | T | 0.23 | 0.22 | Dominant | 1.02 | 0.68-1.52 | 0.927 |  |
|  |  |  |  |  |  | Recessive | 1.22 | 0.53-2.79 | 0.634 |  |
|  |  |  |  |  |  | Additive | 1.04 | 0.75-1.45 | 0.799 |  |
|  |  |  |  |  |  | Allelic |  |  | 0.783 | 0.08 |
|  |  |  |  |  |  |  |  |  |  |  |
| rs9642393 | 7 | 55245647 | C | 0.26 | 0.26 | Dominant | 0.76 | 0.51-1.13 | 0.177 |  |
|  |  |  |  |  |  | Recessive | 0.88 | 0.39-1.97 | 0.757 |  |
|  |  |  |  |  |  | Additive | 0.82 | 0.59.133 | 0.227 |  |
|  |  |  |  |  |  | Allelic |  |  | 0.251 | 1.32 |
|  |  |  |  |  |  |  |  |  |  |  |
| rs2291427 | 10 | 45936224 | A | 0.35 | 0.30 | Dominant | 1.25 | 0.85-1.85 | 0.261 |  |
|  |  |  |  |  |  | Recessive | 1.48 | 0.82-2.65 | 0.189 |  |
|  |  |  |  |  |  | Additive | 1.23 | 0.93-1.64 | 0.148 |  |
|  |  |  |  |  |  | Allelic |  |  | 0.149 | 2.07 |
|  |  |  |  |  |  |  |  |  |  |  |
| rs2031920 | 10 | 135339845 | T | 0.02 | 0.03 | Dominant | 0.62 | 0.21-1.85 | 0.389 |  |
|  |  |  |  |  |  | Recessive | NA | NA | NA |  |
|  |  |  |  |  |  | Additive | 0.62 | 0.21-1.85 | 0.389 |  |
|  |  |  |  |  |  | Allelic |  |  | 0.401 | 0.70 |
|  |  |  |  |  |  |  |  |  |  |  |
| rs1695 | 11 | 67352689 | G | 0.28 | 0.33 | Dominant | 0.78 | 0.53-1.15 | 0.213 |  |
|  |  |  |  |  |  | Recessive | 0.79 | 0.41-1.53 | 0.486 |  |
|  |  |  |  |  |  | Additive | 0.83 | 0.62.11 | 0.212 |  |
|  |  |  |  |  |  | Allelic |  |  | 0.186 | 1.75 |
|  |  |  |  |  |  |  |  |  |  |  |
| rs2682826 | 12 | 117652838 | A | 0.31 | 0.27 | Dominant | 1.29 | 0.87-1.90 | 0.201 |  |
|  |  |  |  |  |  | Recessive | 1.17 | 0.61-2.26 | 0.633 |  |
|  |  |  |  |  |  | Additive | 1.19 | 0.89-1.59 | 0.236 |  |
|  |  |  |  |  |  | Allelic |  |  | 0.215 | 1.54 |
|  |  |  |  |  |  |  |  |  |  |  |
| rs2606345 | 15 | 75017176 | C | 0.32 | 0.36 | Dominant | 0.69 | 0.47-1.03 | 0.067 |  |
|  |  |  |  |  |  | Recessive | 1.03 | 0.59-1.79 | 0.908 |  |
|  |  |  |  |  |  | Additive | 0.84 | 0.63-1.11 | 0.217 |  |
|  |  |  |  |  |  | Allelic |  |  | 0.183 | 1.77 |
|  |  |  |  |  |  |  |  |  |  |  |
| rs1801275 | 16 | 27374400 | G | 0.20 | 0.21 | Dominant | 1.09 | 0.73-1.63 | 0.658 |  |
|  |  |  |  |  |  | Recessive | 0.37 | 0.11-1.25 | 0.109 |  |
|  |  |  |  |  |  | Additive | 0.96 | 0.69-1.34 | 0.801 |  |
|  |  |  |  |  |  | Allelic |  |  | 0.840 | 0.04 |
|  |  |  |  |  |  |  |  |  |  |  |
| rs9303277 | 17 | 37976469 | C | 0.51 | 0.48 | Dominant | 0.91 | 0.59-1.41 | 0.669 |  |
|  |  |  |  |  |  | Recessive | 1.56 | 1.00-2.43 | 0.048 |  |
|  |  |  |  |  |  | Additive | 1.14 | 0.86-1.50 | 0.361 |  |
|  |  |  |  |  |  | Allelic |  |  | 0.328 | 0.96 |
|  |  |  |  |  |  |  |  |  |  |  |
| rs11557467 | 17 | 38028634 | G | 0.49 | 0.49 | Dominant | 0.83 | 0.54-1.28 | 0.404 |  |
|  |  |  |  |  |  | Recessive | 1.31 | 0.84-2.04 | 0.232 |  |
|  |  |  |  |  |  | Additive | 1.03 | 0.78-1.35 | 0.842 |  |
|  |  |  |  |  |  | Allelic |  |  | 0.798 | 0.07 |
|  |  |  |  |  |  |  |  |  |  |  |
| rs8067378 | 17 | 38051348 | A | 0.50 | 0.48 | Dominant | 0.85 | 0.55-1.31 | 0.455 |  |
|  |  |  |  |  |  | Recessive | 1.49 | 0.96-2.33 | 0.075 |  |
|  |  |  |  |  |  | Additive | 1.09 | 0.83-1.44 | 0.548 |  |
|  |  |  |  |  |  | Allelic |  |  | 0.514 | 0.43 |
|  |  |  |  |  |  |  |  |  |  |  |
| rs2290400 | 17 | 38066240 | T | 0.50 | 0.49 | Dominant | 0.83 | 0.54-1.28 | 0.409 |  |
|  |  |  |  |  |  | Recessive | 1.39 | 0.90-2.16 | 0.132 |  |
|  |  |  |  |  |  | Additive | 1.06 | 0.81-1.39 | 0.689 |  |
|  |  |  |  |  |  | Allelic |  |  | 0.661 | 0.19 |
|  |  |  |  |  |  |  |  |  |  |  |
| rs7216389 | 17 | 38069949 | T | 0.5 | 0.48 | Dominant | 0.83 | 0.54-1.29 | 0.412 |  |
|  |  |  |  |  |  | Recessive | 0·51 | 0·17-1·49 | 0·217 |  |
|  |  |  |  |  |  | Additive | 1.06 | 0.80-1.39 | 0.684 |  |
|  |  |  |  |  |  | Allelic |  |  | 0.650 | 0.21 |
|  |  |  |  |  |  |  |  |  |  |  |
| rs1136410 | 1 | 226555302 | G | 0.16 | 0.19 | Dominant | 0.90 | 0.59-1.37 | 0.627 |  |
|  |  |  |  |  |  | Recessive | 0.47 | 0.14-1.59 | 0.222 |  |
|  |  |  |  |  |  | Additive | 0.86 | 0.59-1.23 | 0.396 |  |
|  |  |  |  |  |  | Allelic |  |  | 0.362 | 0.83 |
|  |  |  |  |  |  |  |  |  |  |  |
| rs1047840 | 1 | 242042301 | A | 0.35 | 0.38 | Dominant | 0.77 | 0.52-1.15 | 0.199 |  |
|  |  |  |  |  |  | Recessive | 1.12 | 0.65-1.90 | 0.691 |  |
|  |  |  |  |  |  | Additive | 0.91 | 0.68-1.19 | 0.485 |  |
|  |  |  |  |  |  | Allelic |  |  | 0.469 | 0.52 |
|  |  |  |  |  |  |  |  |  |  |  |
| rs828704 | 2 | 216993611 | C | 0.23 | 0.19 | Dominant | 1.29 | 0.87-1.93 | 0.205 |  |
|  |  |  |  |  |  | Recessive | 1.40 | 0.60-3.26 | 0.434 |  |
|  |  |  |  |  |  | Additive | 1.25 | 0.90-1.73 | 0.181 |  |
|  |  |  |  |  |  | Allelic |  |  | 0.179 | 1.79 |
|  |  |  |  |  |  |  |  |  |  |  |
| rs7721416 | 5 | 82434993 | A | 0.43 | 0.47 | Dominant | 0.77 | 0.51-1.17 | 0.214 |  |
|  |  |  |  |  |  | Recessive | 0.96 | 0.59-1.54 | 0.856 |  |
|  |  |  |  |  |  | Additive | 0.88 | 0.67-1.16 | 0.363 |  |
|  |  |  |  |  |  | Allelic |  |  | 0.388 | 0.74 |
|  |  |  |  |  |  |  |  |  |  |  |
| rs2662242 | 5 | 82484885 | C | 0.45 | 0.48 | Dominant | 0.73 | 0.48-1.12 | 0.147 |  |
|  |  |  |  |  |  | Recessive | 1.07 | 0.67-1.69 | 0.783 |  |
|  |  |  |  |  |  | Additive | 0.89 | 0.68-1.86 | 0.448 |  |
|  |  |  |  |  |  | Allelic |  |  | 0.473 | 0.52 |
|  |  |  |  |  |  |  |  |  |  |  |
| rs16900208 | 5 | 82489315 | G | 0 | 0.00 | Dominant | 2.099e-009 | 0-inf | 0.999 |  |
|  |  |  |  |  |  | Recessive | NA | NA | NA |  |
|  |  |  |  |  |  | Additive | 2.099e-009 | 0-inf | 0.999 |  |
|  |  |  |  |  |  | Allelic |  |  | 0.599 | 0.28 |
|  |  |  |  |  |  |  |  |  |  |  |
| rs13161662 | 5 | 82505596 | G | 0.39 | 0.39 | Dominant | 1.09 | 0.69-1.76 | 0.696 |  |
|  |  |  |  |  |  | Recessive | 0.94 | 0.49-1.79 | 0.859 |  |
|  |  |  |  |  |  | Additive | 1.03 | 0.74-1.43 | 0.855 |  |
|  |  |  |  |  |  | Allelic |  |  | 0.840 | 0.04 |
|  |  |  |  |  |  |  |  |  |  |  |
| rs7715771 | 5 | 82521868 | T | 0.06 | 0.04 | Dominant | 1.54 | 0.83-2.88 | 0.171 |  |
|  |  |  |  |  |  | Recessive | 3.45 | 0.21-56.55 | 0.386 |  |
|  |  |  |  |  |  | Additive | 1.55 | 0.86-2.77 | 0.142 |  |
|  |  |  |  |  |  | Allelic |  |  | 0.145 | 2.12 |
|  |  |  |  |  |  |  |  |  |  |  |
| rs3777015 | 5 | 82648883 | G | 0.03 | 0.04 | Dominant | 0.94 | 0.42-2.11 | 0.881 |  |
|  |  |  |  |  |  | Recessive | NA | NA | NA |  |
|  |  |  |  |  |  | Additive | 0.94 | 0.42-2.11 | 0.881 |  |
|  |  |  |  |  |  | Allelic |  |  | 0.913 | 0.01 |
|  |  |  |  |  |  |  |  |  |  |  |
| rs1805377 | 5 | 82648943 | A | 0.12 | 0.13 | Dominant | 1.00 | 0.63-1.59 | 0.993 |  |
|  |  |  |  |  |  | Recessive | 2.148e-009 | 0-inf | 0.998 |  |
|  |  |  |  |  |  | Additive | 0.89 | 0.59-1.36 | 0.591 |  |
|  |  |  |  |  |  | Allelic |  |  | 0.604 | 0.27 |
|  |  |  |  |  |  |  |  |  |  |  |
| rs1056503 | 5 | 82648977 | G | 0.12 | 0.13 | Dominant | 1.03 | 0.65-1.62 | 0.913 |  |
|  |  |  |  |  |  | Recessive | 2.116e-009 | 0-inf | 0.998 |  |
|  |  |  |  |  |  | Additive | 0.91 | 0.60-1.38 | 0.651 |  |
|  |  |  |  |  |  | Allelic |  |  | 0.667 | 0.19 |
|  |  |  |  |  |  |  |  |  |  |  |
| rs7003908 | 8 | 48770702 | C | 0.33 | 0.35 | Dominant | 0.87 | 0.58-1.29 | 0.478 |  |
|  |  |  |  |  |  | Recessive | 0.89 | 0.48-1.67 | 0.722 |  |
|  |  |  |  |  |  | Additive | 0.9 | 0.67-1.21 | 0.489 |  |
|  |  |  |  |  |  | Allelic |  |  | 0.499 | 0.46 |
|  |  |  |  |  |  |  |  |  |  |  |
| rs12917 | 10 | 131506283 | T | 0.15 | 0.12 | Dominant | 1.43 | 0.92-2.22 | 0.115 |  |
|  |  |  |  |  |  | Recessive | 0.50 | 0.06-4.14 | 0.522 |  |
|  |  |  |  |  |  | Additive | 1.30 | 0.87-1.95 | 0.199 |  |
|  |  |  |  |  |  | Allelic |  |  | 0.233 | 1.42 |
|  |  |  |  |  |  |  |  |  |  |  |
| rs2308321 | 10 | 131565064 | G | 0.14 | 0.13 | Dominant | 1.15 | 0.74-1.79 | 0.532 |  |
|  |  |  |  |  |  | Recessive | 0.42 | 0.05-3.34 | 0.410 |  |
|  |  |  |  |  |  | Additive | 1.07 | 0.72-1.61 | 0.730 |  |
|  |  |  |  |  |  | Allelic |  |  | 0.715 | 0.13 |
|  |  |  |  |  |  |  |  |  |  |  |
| rs228599 | 11 | 108107660 | G | 0.45 | 0.45 | Dominant | 1.11 | 0.73-1.69 | 0.624 |  |
|  |  |  |  |  |  | Recessive | 0.89 | 0.55-1.46 | 0.657 |  |
|  |  |  |  |  |  | Additive | 1.01 | 0.77-1.32 | 0.949 |  |
|  |  |  |  |  |  | Allelic |  |  | 0.928 | 0.01 |
|  |  |  |  |  |  |  |  |  |  |  |
| rs3092992 | 11 | 108195779 | C | 0.05 | 0.05 | Dominant | 1.09 | 0.56-2.09 | 0.801 |  |
|  |  |  |  |  |  | Recessive | 2.108e-009 | 0-inf | 0.999 |  |
|  |  |  |  |  |  | Additive | 1.03 | 0.55-1.93 | 0.925 |  |
|  |  |  |  |  |  | Allelic |  |  | 0.931 | 0.01 |
|  |  |  |  |  |  |  |  |  |  |  |
| rs664143 | 11 | 108225661 | A | 0.45 | 0.45 | Dominant | 1.10 | 0.73-1.68 | 0.644 |  |
|  |  |  |  |  |  | Recessive | 0.88 | 0.54-1.43 | 0.605 |  |
|  |  |  |  |  |  | Additive | 1.00 | 0.77-1.31 | 0.996 |  |
|  |  |  |  |  |  | Allelic |  |  | 0.994 | 6.285e-005 |
|  |  |  |  |  |  |  |  |  |  |  |
| rs170548 | 11 | 108234836 | C | 0.32 | 0.31 | Dominant | 1.00 | 0.68-1.48 | 0.988 |  |
|  |  |  |  |  |  | Recessive | 1.17 | 0.64-2.13 | 0.607 |  |
|  |  |  |  |  |  | Additive | 1.04 | 0.78-1.38 | 0.803 |  |
|  |  |  |  |  |  | Allelic |  |  | 0.764 | 0.09 |
|  |  |  |  |  |  |  |  |  |  |  |
| rs3092993 | 11 | 108235115 | A | 0.12 | 0.14 | Dominant | 0.83 | 0.53-1.30 | 0.414 |  |
|  |  |  |  |  |  | Recessive | 0.61 | 0.07-5.16 | 0.654 |  |
|  |  |  |  |  |  | Additive | 0.83 | 0.54-1.27 | 0.387 |  |
|  |  |  |  |  |  | Allelic |  |  | 0.382 | 0.76 |
|  |  |  |  |  |  |  |  |  |  |  |
| rs3093739 | 13 | 108867401 | G | 0.11 | 0.14 | Dominant | 0.72 | 0.45-1.16 | 0.174 |  |
|  |  |  |  |  |  | Recessive | 1.44 | 0.37-5.60 | 0.596 |  |
|  |  |  |  |  |  | Additive | 0.79 | 0.51-1.21 | 0.281 |  |
|  |  |  |  |  |  | Allelic |  |  | 0.258 | 1.28 |
|  |  |  |  |  |  |  |  |  |  |  |
| rs3093737 | 13 | 108867483 | C | 0.09 | 0.09 | Dominant | 0.89 | 0.52-1.52 | 0.667 |  |
|  |  |  |  |  |  | Recessive | 2.90 | 0.63-13.39 | 0.172 |  |
|  |  |  |  |  |  | Additive | 0.99 | 0.62-1.60 | 0.983 |  |
|  |  |  |  |  |  | Allelic |  |  | 0.913 | 0.01 |
|  |  |  |  |  |  |  |  |  |  |  |
| rs3212092 | 14 | 104168644 | A | 0.02 | 0.01 | Dominant | 2.55 | 0.70-9.23 | 0.154 |  |
|  |  |  |  |  |  | Recessive | NA | NA | NA |  |
|  |  |  |  |  |  | Additive | 2.55 | 0.70-9.23 | 0.154 |  |
|  |  |  |  |  |  | Allelic |  |  | 0.153 | 2.04 |
|  |  |  |  |  |  |  |  |  |  |  |
| rs861530 | 14 | 104174123 | T | 0.26 | 0.30 | Dominant | 0.81 | 0.52-1.25 | 0.336 |  |
|  |  |  |  |  |  | Recessive | 0.75 | 0.35-1.59 | 0.445 |  |
|  |  |  |  |  |  | Additive | 0.84 | 0.6-1.165 | 0.29 |  |
|  |  |  |  |  |  | Allelic |  |  | 0.266 | 1.24 |
|  |  |  |  |  |  |  |  |  |  |  |
| rs1625895 | 17 | 7578115 | T | 0.11 | 0.13 | Dominant | 0.78 | 0.49-1.25 | 0.307 |  |
|  |  |  |  |  |  | Recessive | 0.49 | 0.06-4.11 | 0.517 |  |
|  |  |  |  |  |  | Additive | 0.79 | 0.51-1.21 | 0.271 |  |
|  |  |  |  |  |  | Allelic |  |  | 0.285 | 1.15 |
|  |  |  |  |  |  |  |  |  |  |  |
| rs2287499 | 17 | 7592168 | G | 0.15 | 0.14 | Dominant | 1.17 | 0.76-1.81 | 0.476 |  |
|  |  |  |  |  |  | Recessive | 0.64 | 0.14-2.98 | 0.573 |  |
|  |  |  |  |  |  | Additive | 1.09 | 0.74-1.62 | 0.641 |  |
|  |  |  |  |  |  | Allelic |  |  | 0.611 | 0.26 |
|  |  |  |  |  |  |  |  |  |  |  |
| rs12450550 | 17 | 48456193 | C | 0.28 | 0.28 | Dominant | 1.13 | 0.77-1.67 | 0.538 |  |
|  |  |  |  |  |  | Recessive | 0.47 | 0.19-1.14 | 0.095 |  |
|  |  |  |  |  |  | Additive | 0.96 | 0.71-1.31 | 0.802 |  |
|  |  |  |  |  |  | Allelic |  |  | 0.816 | 0.05 |
|  |  |  |  |  |  |  |  |  |  |  |
| rs105038 | 19 | 4414710 | T | 0.23 | 0.29 | Dominant | 0.69 | 0.46-1.02 | 0.062 |  |
|  |  |  |  |  |  | Recessive | 0.70 | 0.33-1.47 | 0.348 |  |
|  |  |  |  |  |  | Additive | 0.75 | 0.55-1.02 | 0.066 |  |
|  |  |  |  |  |  | Allelic |  |  | 0.061 | 3.51 |
|  |  |  |  |  |  |  |  |  |  |  |
| rs243356 | 19 | 4415452 | T | 0.20 | 0.24 | Dominant | 0.75 | 0.49-1.12 | 0.155 |  |
|  |  |  |  |  |  | Recessive | 0.74 | 0.34-1.63 | 0.452 |  |
|  |  |  |  |  |  | Additive | 0.79 | 0.58-1.09 | 0.158 |  |
|  |  |  |  |  |  | Allelic |  |  | 0.136 | 2.22 |
|  |  |  |  |  |  |  |  |  |  |  |
| rs13181 | 19 | 45854919 | G | 0.36 | 0.36 | Dominant | 1.14 | 0.76-1.7 | 0.516 |  |
|  |  |  |  |  |  | Recessive | 0.76 | 0.40-1.45 | 0.408 |  |
|  |  |  |  |  |  | Additive | 1.01 | 0.76-1.35 | 0.939 |  |
|  |  |  |  |  |  | Allelic |  |  | 0.925 | 0.01 |
|  |  |  |  |  |  |  |  |  |  |  |
| rs238406 | 19 | 45868309 | T | 0.44 | 0.45 | Dominant | 0.99 | 0.65-1.53 | 0.982 |  |
|  |  |  |  |  |  | Recessive | 0.93 | 0.56-1.56 | 0.787 |  |
|  |  |  |  |  |  | Additive | 0.98 | 0.73-1.30 | 0.866 |  |
|  |  |  |  |  |  | Allelic |  |  | 0.872 | 0.03 |
|  |  |  |  |  |  |  |  |  |  |  |
| rs1035938 | 19 | 48183771 | T | 0.27 | 0.24 | Dominant | 1.29 | 0.87-1.90 | 0.201 |  |
|  |  |  |  |  |  | Recessive | 0.82 | 0.35-1.92 | 0.652 |  |
|  |  |  |  |  |  | Additive | 1.15 | 0.84-1.57 | 0.389 |  |
|  |  |  |  |  |  | Allelic |  |  | 0.363 | 0.83 |
|  |  |  |  |  |  |  |  |  |  |  |
| rs20579 | 19 | 48668830 | A | 0.09 | 0.13 | Dominant | 0.66 | 0.39-1.08 | 0.097 |  |
|  |  |  |  |  |  | Recessive | 2.134e-009 | 0-inf | 0.999 |  |
|  |  |  |  |  |  | Additive | 0.64 | 0.39-1.04 | 0.070 |  |
|  |  |  |  |  |  | Allelic |  |  | 0.070 | 3.27 |
|  |  |  |  |  |  |  |  |  |  |  |
| rs132771 | 22 | 42025350 | A | 0.14 | 0.16 | Dominant | 0.90 | 0.58-1.39 | 0.639 |  |
|  |  |  |  |  |  | Recessive | 2.356e-009 | 0-inf | 0.998 |  |
|  |  |  |  |  |  | Additive | 0.83 | 0.56-1.25 | 0.383 |  |
|  |  |  |  |  |  | Allelic |  |  | 0.429 | 0.62 |

***a***: MAF=Minor Allele Frequency ***b*:** OR adjusted for age, sex, and country
