# Supplementary material for: Common genetic variations in cell cycle and DNA repair pathways associated with pediatric brain tumor susceptibility
Source: Oncotarget. 2016 Aug 24;7(39):63640–50. doi: 10.18632/oncotarget.11575 (PMC5325391; doi:10.18632/oncotarget.11575)
Supplement: Supplementary file 4 [file oncotarget-07-63640-s004.doc]

**Table S3. Summary results for SNPs unassociated with non-astrocytoma subtypes**

| **SNP** | **Chr.** | **Location (bp)** | **Minor allele** | **MAF*^a^* in cases** | **MAF*^a^* in controls** | **Model** | **OR*^b^*** | **95% CI** | **P** | **CHISQ** |
| --- | --- | --- | --- | --- | --- | --- | --- | --- | --- | --- |
|  |  |  |  |  |  |  |  |  |  |  |
| rs1801131 | 1 | 11854476 | G | 0.31 | 0.32 | Dominant | 0.89 | 0.58-1.37 | 0.598 |  |
|  |  |  |  |  |  | Recessive | 0.90 | 0.44-1.82 | 0.774 |  |
|  |  |  |  |  |  | Additive | 0.92 | 0.66-1.27 | 0.598 |  |
|  |  |  |  |  |  | Allelic |  |  | 0.807 | 0.06 |
|  |  |  |  |  |  |  |  |  |  |  |
| rs1801133 | 1 | 11856378 | A | 0.33 | 0.28 | Dominant | 1.42 | 0.93-2.17 | 0.108 |  |
|  |  |  |  |  |  | Recessive | 1.46 | 0.73-2.94 | 0.286 |  |
|  |  |  |  |  |  | Additive | 1.32 | 0.96-1.83 | 0.088 |  |
|  |  |  |  |  |  | Allelic |  |  | 0.195 | 1.68 |
|  |  |  |  |  |  |  |  |  |  |  |
| rs3917727 | 1 | 169581258 | G | 0.31 | 0.35 | Dominant | 0.67 | 0.44-1.03 | 0.068 |  |
|  |  |  |  |  |  | Recessive | 1.09 | 0.59-2.03 | 0.782 |  |
|  |  |  |  |  |  | Additive | 0.83 | 0.60-1.13 | 0.238 |  |
|  |  |  |  |  |  | Allelic |  |  | 0.178 | 1.81 |
|  |  |  |  |  |  |  |  |  |  |  |
| rs11579965 | 1 | 183563161 | G | 0.06 | 0.08 | Dominant | 0.78 | 0.41-1.48 | 0.453 |  |
|  |  |  |  |  |  | Recessive | 3.052e-009 | 0-inf | 0.999 |  |
|  |  |  |  |  |  | Additive | 0.76 | 0.41-1.42 | 0.390 |  |
|  |  |  |  |  |  | Allelic |  |  | 0.385 | 0.76 |
|  |  |  |  |  |  |  |  |  |  |  |
| rs1800896 | 1 | 206946897 | C | 0.43 | 0.47 | Dominant | 0.92 | 0.59-1.46 | 0.753 |  |
|  |  |  |  |  |  | Recessive | 0.66 | 0.39-1.14 | 0.139 |  |
|  |  |  |  |  |  | Additive | 0.85 | 0.64-1.14 | 0.289 |  |
|  |  |  |  |  |  | Allelic |  |  | 0.227 | 1.46 |
|  |  |  |  |  |  |  |  |  |  |  |
| rs1805087 | 1 | 237048500 | G | 0.19 | 0.18 | Dominant | 1.12 | 0.72-1.75 | 0.624 |  |
|  |  |  |  |  |  | Recessive | 1.34 | 0.51-3.52 | 0.552 |  |
|  |  |  |  |  |  | Additive | 1.12 | 0.78-1.62 | 0.535 |  |
|  |  |  |  |  |  | Allelic |  |  | 0.564 | 0.33 |
|  |  |  |  |  |  |  |  |  |  |  |
| rs1045485 | 2 | 202149589 | C | 0.12 | 0.12 | Dominant | 0.89 | 0.53-1.47 | 0.638 |  |
|  |  |  |  |  |  | Recessive | 3.54 | 0.75-16.78 | 0.111 |  |
|  |  |  |  |  |  | Additive | 0.99 | 0.62-1.57 | 0.971 |  |
|  |  |  |  |  |  | Allelic |  |  | 0.986 | 0.00 |
|  |  |  |  |  |  |  |  |  |  |  |
| rs1801394 | 5 | 7870973 | A | 0.42 | 0.46 | Dominant | 0.90 | 0.57-1.44 | 0.667 |  |
|  |  |  |  |  |  | Recessive | 0.64 | 0.36-1.15 | 0.133 |  |
|  |  |  |  |  |  | Additive | 0.83 | 0.61-1.14 | 0.248 |  |
|  |  |  |  |  |  | Allelic |  |  | 0.294 | 1.10 |
|  |  |  |  |  |  |  |  |  |  |  |
| rs1800925 | 5 | 131992809 | T | 0.23 | 0.20 | Dominant | 1.31 | 0.85-2.02 | 0.219 |  |
|  |  |  |  |  |  | Recessive | 0.37 | 0.08-1.63 | 0.189 |  |
|  |  |  |  |  |  | Additive | 1.11 | 0.77-1.61 | 0.568 |  |
|  |  |  |  |  |  | Allelic |  |  | 0.406 | 0.69 |
|  |  |  |  |  |  |  |  |  |  |  |
| rs20541 | 5 | 131995964 | A | 0.22 | 0.22 | Dominant | 0.94 | 0.61-1.45 | 0.783 |  |
|  |  |  |  |  |  | Recessive | 0.81 | 0.33-2.02 | 0.653 |  |
|  |  |  |  |  |  | Additive | 0.93 | 0.66-1.32 | 0.690 |  |
|  |  |  |  |  |  | Allelic |  |  | 0.799 | 0.06 |
|  |  |  |  |  |  |  |  |  |  |  |
| rs4947979 | 7 | 55195625 | G | 0.21 | 0.18 | Dominant | 1.25 | 0.81-1.93 | 0.322 |  |
|  |  |  |  |  |  | Recessive | 1.2 | 0.33-4.37 | 0.782 |  |
|  |  |  |  |  |  | Additive | 1.21 | 0.82-1.78 | 0.334 |  |
|  |  |  |  |  |  | Allelic |  |  | 0.363 | 0.83 |
|  |  |  |  |  |  |  |  |  |  |  |
| rs11506105 | 7 | 55220177 | A | 0.40 | 0.45 | Dominant | 0.69 | 0.45-1.07 | 0.096 |  |
|  |  |  |  |  |  | Recessive | 0.86 | 0.50-1.45 | 0.564 |  |
|  |  |  |  |  |  | Additive | 0.81 | 0.60-1.09 | 0.159 |  |
|  |  |  |  |  |  | Allelic |  |  | 0.232 | 1.43 |
|  |  |  |  |  |  |  |  |  |  |  |
| rs4947986 | 7 | 55221655 | A | 0.25 | 0.29 | Dominant | 0.86 | 0.56-1.31 | 0.479 |  |
|  |  |  |  |  |  | Recessive | 0.46 | 0.16-1.19 | 0.110 |  |
|  |  |  |  |  |  | Additive | 0.80 | 0.58-1.13 | 0.210 |  |
|  |  |  |  |  |  | Allelic |  |  | 0.248 | 1.33 |
|  |  |  |  |  |  |  |  |  |  |  |
| rs3752651 | 7 | 55229543 | C | 0.19 | 0.21 | Dominant | 0.86 | 0.54-1.36 | 0.514 |  |
|  |  |  |  |  |  | Recessive | 0.97 | 0.35-2.65 | 0.951 |  |
|  |  |  |  |  |  | Additive | 0.89 | 0.62-1.31 | 0.576 |  |
|  |  |  |  |  |  | Allelic |  |  | 0.598 | 0.28 |
|  |  |  |  |  |  |  |  |  |  |  |
| rs1468727 | 7 | 55230105 | T | 0.25 | 0.22 | Dominant | 1.06 | 0.69-1.63 | 0.800 |  |
|  |  |  |  |  |  | Recessive | 2.01 | 0.91-4.46 | 0.084 |  |
|  |  |  |  |  |  | Additive | 1.17 | 0.83-1.65 | 0.378 |  |
|  |  |  |  |  |  | Allelic |  |  | 0.402 | 0.70 |
|  |  |  |  |  |  |  |  |  |  |  |
| rs2291427 | 10 | 45936224 | A | 0.28 | 0.31 | Dominant | 0.91 | 0.59-1.38 | 0.649 |  |
|  |  |  |  |  |  | Recessive | 0.84 | 0.39-1.78 | 0.641 |  |
|  |  |  |  |  |  | Additive | 0.91 | 0.66-1.26 | 0.579 |  |
|  |  |  |  |  |  | Allelic |  |  | 0.539 | 0.38 |
|  |  |  |  |  |  |  |  |  |  |  |
| rs2031920 | 10 | 135339845 | T | 0.01 | 0.03 | Dominant | 0.19 | 0.02-1.42 | 0.105 |  |
|  |  |  |  |  |  | Recessive | NA | NA | NA |  |
|  |  |  |  |  |  | Additive | 0.19 | 0.02-1.42 | 0.105 |  |
|  |  |  |  |  |  | Allelic |  |  | 0.062 | 3.48 |
|  |  |  |  |  |  |  |  |  |  |  |
| rs1695 | 11 | 67352689 | G | 0.36 | 0.33 | Dominant | 1.54 | 0.99-2.39 | 0.052 |  |
|  |  |  |  |  |  | Recessive | 0.77 | 0.36-1.62 | 0.486 |  |
|  |  |  |  |  |  | Additive | 1.21 | 0.88-1.66 | 0.248 |  |
|  |  |  |  |  |  | Allelic |  |  | 0.337 | 0.92 |
|  |  |  |  |  |  |  |  |  |  |  |
| rs2682826 | 12 | 117652838 | A | 0.25 | 0.27 | Dominant | 1.03 | 0.67-1.57 | 0.898 |  |
|  |  |  |  |  |  | Recessive | 0.55 | 0.21-1.44 | 0.221 |  |
|  |  |  |  |  |  | Additive | 0.93 | 0.66-1.31 | 0.676 |  |
|  |  |  |  |  |  | Allelic |  |  | 0.663 | 0.19 |
|  |  |  |  |  |  |  |  |  |  |  |
| rs2606345 | 15 | 75017176 | C | 0.35 | 0.36 | Dominant | 1.02 | 0.67-1.56 | 0.929 |  |
|  |  |  |  |  |  | Recessive | 0.78 | 0.41-1.48 | 0.444 |  |
|  |  |  |  |  |  | Additive | 0.95 | 0.70-1.29 | 0.749 |  |
|  |  |  |  |  |  | Allelic |  |  | 0.716 | 0.13 |
|  |  |  |  |  |  |  |  |  |  |  |
| rs1801275 | 16 | 27374400 | G | 0.19 | 0.21 | Dominant | 0.89 | 0.58-1.39 | 0.633 |  |
|  |  |  |  |  |  | Recessive | 0.71 | 0.26-1.8 | 0.489 |  |
|  |  |  |  |  |  | Additive | 0.89 | 0.62-1.27 | 0.514 |  |
|  |  |  |  |  |  | Allelic |  |  | 0.583 | 0.30 |
|  |  |  |  |  |  |  |  |  |  |  |
| rs9303277 | 17 | 37976469 | C | 0.49 | 0.48 | Dominant | 1.24 | 0.75-2.06 | 0.398 |  |
|  |  |  |  |  |  | Recessive | 0.89 | 0.53-1.51 | 0.666 |  |
|  |  |  |  |  |  | Additive | 1.04 | 0.77-1.42 | 0.783 |  |
|  |  |  |  |  |  | Allelic |  |  | 0.722 | 0.13 |
|  |  |  |  |  |  |  |  |  |  |  |
| rs11557467 | 17 | 38028634 | G | 0.50 | 0.49 | Dominant | 1.26 | 0.76-2.09 | 0.364 |  |
|  |  |  |  |  |  | Recessive | 0.93 | 0.56-1.53 | 0.762 |  |
|  |  |  |  |  |  | Additive | 1.06 | 0.78-1.43 | 0.703 |  |
|  |  |  |  |  |  | Allelic |  |  | 0.643 | 0.22 |
|  |  |  |  |  |  |  |  |  |  |  |
| rs8067378 | 17 | 38051348 | A | 0.5 | 0.49 | Dominant | 1.18 | 0.71-1.96 | 0.523 |  |
|  |  |  |  |  |  | Recessive | 1.00 | 0.59-1.67 | 0.995 |  |
|  |  |  |  |  |  | Additive | 1.07 | 0.78-1.45 | 0.685 |  |
|  |  |  |  |  |  | Allelic |  |  | 0.615 | 0.25 |
|  |  |  |  |  |  |  |  |  |  |  |
| rs2290400 | 17 | 38066240 | T | 0.50 | 0.49 | Dominant | 1.2 | 0.73-1.99 | 0.479 |  |
|  |  |  |  |  |  | Recessive | 0.95 | 0.58-1.57 | 0.847 |  |
|  |  |  |  |  |  | Additive | 1.05 | 0.78-1.43 | 0.747 |  |
|  |  |  |  |  |  | Allelic |  |  | 0.669 | 0.18 |
|  |  |  |  |  |  |  |  |  |  |  |
| rs7216389 | 17 | 38069949 | T | 0.49 | 0.48 | Dominant | 1.17 | 0.70-1.93 | 0.553 |  |
|  |  |  |  |  |  | Recessive | 0.85 | 0.50-1.44 | 0.549 |  |
|  |  |  |  |  |  | Additive | 1 | 0.73-1.37 | 0.998 |  |
|  |  |  |  |  |  | Allelic |  |  | 0.862 | 0.03 |
|  |  |  |  |  |  |  |  |  |  |  |
| rs1136410 | 1 | 226555302 | G | 0.16 | 0.19 | Dominant | 0.78 | 0.48-1.25 | 0.303 |  |
|  |  |  |  |  |  | Recessive | 0.85 | 0.31-2.33 | 0.752 |  |
|  |  |  |  |  |  | Additive | 0.83 | 0.56-1.22 | 0.345 |  |
|  |  |  |  |  |  | Allelic |  |  | 0.329 | 0.95 |
|  |  |  |  |  |  |  |  |  |  |  |
| rs1047840 | 1 | 242042301 | A | 0.37 | 0.38 | Dominant | 0.96 | 0.63-1.49 | 0.868 |  |
|  |  |  |  |  |  | Recessive | 0.91 | 0.491.68 | 0.768 |  |
|  |  |  |  |  |  | Additive | 0.96 | 0.71-1.30 | 0.789 |  |
|  |  |  |  |  |  | Allelic |  |  | 0.817 | 0.05 |
|  |  |  |  |  |  |  |  |  |  |  |
| rs828704 | 2 | 216993611 | C | 0.21 | 0.19 | Dominant | 1 | 0.64-1.55 | 0.999 |  |
|  |  |  |  |  |  | Recessive | 0.98 | 0.382.54 | 0.965 |  |
|  |  |  |  |  |  | Additive | 0.99 | 0.69-1.43 | 0.988 |  |
|  |  |  |  |  |  | Allelic |  |  | 0.686 | 0.16 |
|  |  |  |  |  |  |  |  |  |  |  |
| rs16900208 | 5 | 82489315 | G | 0 | 0.00 | Dominant | 1.596e-009 | 0-inf | 0.999 |  |
|  |  |  |  |  |  | Recessive | NA | NA | NA |  |
|  |  |  |  |  |  | Additive | 1.596e-009 | 0-inf | 0.999 |  |
|  |  |  |  |  |  | Allelic |  |  | 0.629 | 0.23 |
|  |  |  |  |  |  |  |  |  |  |  |
| rs13161662 | 5 | 82505596 | G | 0.43 | 0.39 | Dominant | 1.30 | 0.77-2.19 | 0.319 |  |
|  |  |  |  |  |  | Recessive | 1.23 | 0.65-2.35 | 0.525 |  |
|  |  |  |  |  |  | Additive | 1.20 | 0.85-1.71 | 0.304 |  |
|  |  |  |  |  |  | Allelic |  |  | 0.301 | 1.07 |
|  |  |  |  |  |  |  |  |  |  |  |
| rs7715771 | 5 | 82521868 | T | 0.05 | 0.04 | Dominant | 1.24 | 0.61-2.55 | 0.551 |  |
|  |  |  |  |  |  | Recessive | 1.594e-009 | 0-inf | 0.999 |  |
|  |  |  |  |  |  | Additive | 1.18 | 0.59-2.37 | 0.638 |  |
|  |  |  |  |  |  | Allelic |  |  | 0.645 | 0.21 |
|  |  |  |  |  |  |  |  |  |  |  |
| rs3777015 | 5 | 82648883 | G | 0.06 | 0.04 | Dominant | 1.63 | 0.79-3.33 | 0.183 |  |
|  |  |  |  |  |  | Recessive | NA | NA | NA |  |
|  |  |  |  |  |  | Additive | 1.63 | 0.79-3.33 | 0.183 |  |
|  |  |  |  |  |  | Allelic |  |  | 0.141 | 2.17 |
|  |  |  |  |  |  |  |  |  |  |  |
| rs1805377 | 5 | 82648943 | A | 0.14 | 0.13 | Dominant | 1.08 | 0.66-1.76 | 0.772 |  |
|  |  |  |  |  |  | Recessive | 0.99 | 0.27-3.67 | 0.985 |  |
|  |  |  |  |  |  | Additive | 1.05 | 0.69-1.6 | 0.809 |  |
|  |  |  |  |  |  | Allelic |  |  | 0.649 | 0.21 |
|  |  |  |  |  |  |  |  |  |  |  |
| rs1056503 | 5 | 82648977 | G | 0.15 | 0.13 | Dominant | 1.20 | 0.74-1.95 | 0.461 |  |
|  |  |  |  |  |  | Recessive | 0.99 | 0.26-3.67 | 0.982 |  |
|  |  |  |  |  |  | Additive | 1.14 | 0.75-1.72 | 0.533 |  |
|  |  |  |  |  |  | Allelic |  |  | 0.431 | 0.62 |
|  |  |  |  |  |  |  |  |  |  |  |
| rs7003908 | 8 | 48770702 | C | 0.33 | 0.35 | Dominant | 0.89 | 0.58-1.37 | 0.602 |  |
|  |  |  |  |  |  | Recessive | 0.73 | 0.36-1.50 | 0.396 |  |
|  |  |  |  |  |  | Additive | 0.88 | 0.63-1.21 | 0.426 |  |
|  |  |  |  |  |  | Allelic |  |  | 0.462 | 0.55 |
|  |  |  |  |  |  |  |  |  |  |  |
| rs12917 | 10 | 131506283 | T | 0.14 | 0.12 | Dominant | 1.29 | 0.79-2.09 | 0.298 |  |
|  |  |  |  |  |  | Recessive | 1.25 | 0.25-6.25 | 0.784 |  |
|  |  |  |  |  |  | Additive | 1.25 | 0.81-1.93 | 0.31 |  |
|  |  |  |  |  |  | Allelic |  |  | 0.367 | 0.81 |
|  |  |  |  |  |  |  |  |  |  |  |
| rs2308321 | 10 | 131565064 | G | 0.11 | 0.13 | Dominant | 0.88 | 0.53-1.47 | 0.632 |  |
|  |  |  |  |  |  | Recessive | 2.973e-009 | 0-inf | 0.998 |  |
|  |  |  |  |  |  | Additive | 0.83 | 0.51-1.33 | 0.431 |  |
|  |  |  |  |  |  | Allelic |  |  | 0.345 | 0.89 |
|  |  |  |  |  |  |  |  |  |  |  |
| rs228599 | 11 | 108107660 | G | 0.44 | 0.45 | Dominant | 1.17 | 0.73-1.86 | 0.515 |  |
|  |  |  |  |  |  | Recessive | 0.66 | 0.38-1.18 | 0.159 |  |
|  |  |  |  |  |  | Additive | 0.94 | 0.69-1.27 | 0.705 |  |
|  |  |  |  |  |  | Allelic |  |  | 0.747 | 0.10 |
|  |  |  |  |  |  |  |  |  |  |  |
| rs3092992 | 11 | 108195779 | C | 0.04 | 0.05 | Dominant | 0.89 | 0.42-1.93 | 0.783 |  |
|  |  |  |  |  |  | Recessive | 2.307e-009 | 0-inf | 0.999 |  |
|  |  |  |  |  |  | Additive | 0.86 | 0.41-1.78 | 0.684 |  |
|  |  |  |  |  |  | Allelic |  |  | 0.696 | 0.15 |
|  |  |  |  |  |  |  |  |  |  |  |
| rs664143 | 11 | 108225661 | A | 0.43 | 0.45 | Dominant | 1.17 | 0.74-1.85 | 0.506 |  |
|  |  |  |  |  |  | Recessive | 0.65 | 0.37-1.15 | 0.137 |  |
|  |  |  |  |  |  | Additive | 0.94 | 0.69-1.26 | 0.681 |  |
|  |  |  |  |  |  | Allelic |  |  | 0.692 | 0.16 |
|  |  |  |  |  |  |  |  |  |  |  |
| rs3092993 | 11 | 108235115 | A | 0.12 | 0.14 | Dominant | 0.82 | 0.49-1.34 | 0.429 |  |
|  |  |  |  |  |  | Recessive | 1.82 | 0.35-9.53 | 0.477 |  |
|  |  |  |  |  |  | Additive | 0.87 | 0.55-1.39 | 0.568 |  |
|  |  |  |  |  |  | Allelic |  |  | 0.448 | 0.58 |
|  |  |  |  |  |  |  |  |  |  |  |
| rs3093739 | 13 | 108867401 | G | 0.15 | 0.14 | Dominant | 1.22 | 0.76-1.94 | 0.410 |  |
|  |  |  |  |  |  | Recessive | 0.57 | 0.07-4.65 | 0.596 |  |
|  |  |  |  |  |  | Additive | 1.15 | 0.75-1.76 | 0.536 |  |
|  |  |  |  |  |  | Allelic |  |  | 0.654 | 0.20 |
|  |  |  |  |  |  |  |  |  |  |  |
| rs3093737 | 13 | 108867483 | C | 0.09 | 0.09 | Dominant | 1.14 | 0.65-1.99 | 0.659 |  |
|  |  |  |  |  |  | Recessive | 2.909e-009 | 0-inf | 0.999 |  |
|  |  |  |  |  |  | Additive | 1.06 | 0.62-1.82 | 0.822 |  |
|  |  |  |  |  |  | Allelic |  |  | 0.982 | 0.00 |
|  |  |  |  |  |  |  |  |  |  |  |
| rs3212092 | 14 | 104168644 | A | 0.01 | 0.01 | Dominant | 2.54 | 0.60-10.7 | 0.206 |  |
|  |  |  |  |  |  | Recessive | NA | NA | NA |  |
|  |  |  |  |  |  | Additive | 2.54 | 0.60-10.7 | 0.206 |  |
|  |  |  |  |  |  | Allelic |  |  | 0.253 | 1.31 |
|  |  |  |  |  |  |  |  |  |  |  |
| rs861530 | 14 | 104174123 | T | 0.25 | 0.30 | Dominant | 0.62 | 0.38-1.02 | 0.059 |  |
|  |  |  |  |  |  | Recessive | 1.01 | 0.48-2.14 | 0.971 |  |
|  |  |  |  |  |  | Additive | 0.78 | 0.54-1.12 | 0.177 |  |
|  |  |  |  |  |  | Allelic |  |  | 0.155 | 2.02 |
|  |  |  |  |  |  |  |  |  |  |  |
| rs1625895 | 17 | 7578115 | T | 0.16 | 0.13 | Dominant | 1.28 | 0.80-2.04 | 0.299 |  |
|  |  |  |  |  |  | Recessive | 1.96 | 0.49-7.84 | 0.345 |  |
|  |  |  |  |  |  | Additive | 1.29 | 0.85-1.96 | 0.232 |  |
|  |  |  |  |  |  | Allelic |  |  | 0.271 | 1.21 |
|  |  |  |  |  |  |  |  |  |  |  |
| rs2287499 | 17 | 7592168 | G | 0.10 | 0.14 | Dominant | 0.60 | 0.35-1.04 | 0.067 |  |
|  |  |  |  |  |  | Recessive | 0.64 | 0.14-2.98 | 0.569 |  |
|  |  |  |  |  |  | Additive | 0.65 | 0.40-1.05 | 0.078 |  |
|  |  |  |  |  |  | Allelic |  |  | 0.158 | 1.99 |
|  |  |  |  |  |  |  |  |  |  |  |
| rs243341 | 19 | 4405106 | C | 0.29 | 0.29 | Dominant | 1.12 | 0.73-1.70 | 0.605 |  |
|  |  |  |  |  |  | Recessive | 0.52 | 0.22-1.27 | 0.153 |  |
|  |  |  |  |  |  | Additive | 0.96 | 0.69-1.33 | 0.812 |  |
|  |  |  |  |  |  | Allelic |  |  | 0.868 | 0.03 |
|  |  |  |  |  |  |  |  |  |  |  |
| rs105038 | 19 | 4414710 | T | 0.29 | 0.29 | Dominant | 1.20 | 0.78-1.84 | 0.398 |  |
|  |  |  |  |  |  | Recessive | 0.55 | 0.22-1.33 | 0.183 |  |
|  |  |  |  |  |  | Additive | 1.01 | 0.73-1.40 | 0.950 |  |
|  |  |  |  |  |  | Allelic |  |  | 0.945 | 0.00 |
|  |  |  |  |  |  |  |  |  |  |  |
| rs243356 | 19 | 4415452 | T | 0.26 | 0.24 | Dominant | 1.28 | 0.84-1.96 | 0.256 |  |
|  |  |  |  |  |  | Recessive | 0.53 | 0.20-1.39 | 0.200 |  |
|  |  |  |  |  |  | Additive | 1.06 | 0.76-1.48 | 0.728 |  |
|  |  |  |  |  |  | Allelic |  |  | 0.656 | 0.19 |
|  |  |  |  |  |  |  |  |  |  |  |
| rs2992 | 19 | 4443046 | C | 0.29 | 0.29 | Dominant | 1.16 | 0.76-1.77 | 0.488 |  |
|  |  |  |  |  |  | Recessive | 0.53 | 0.22-1.28 | 0.158 |  |
|  |  |  |  |  |  | Additive | 0.99 | 0.71-1.36 | 0.928 |  |
|  |  |  |  |  |  | Allelic |  |  | 0.947 | 0.00 |
|  |  |  |  |  |  |  |  |  |  |  |
| rs25487 | 19 | 44055726 | T | 0.34 | 0.36 | Dominant | 0.90 | 0.59-1.38 | 0.638 |  |
|  |  |  |  |  |  | Recessive | 0.82 | 0.42-1.59 | 0.556 |  |
|  |  |  |  |  |  | Additive | 0.90 | 0.66-1.24 | 0.527 |  |
|  |  |  |  |  |  | Allelic |  |  | 0.562 | 0.34 |
|  |  |  |  |  |  |  |  |  |  |  |
| rs13181 | 19 | 45854919 | G | 0.38 | 0.36 | Dominant | 1.19 | 0.77-1.85 | 0.421 |  |
|  |  |  |  |  |  | Recessive | 1.04 | 0.55-1.95 | 0.910 |  |
|  |  |  |  |  |  | Additive | 1.11 | 0.81-1.51 | 0.519 |  |
|  |  |  |  |  |  | Allelic |  |  | 0.487 | 0.48 |
|  |  |  |  |  |  |  |  |  |  |  |
| rs238406 | 19 | 45868309 | T | 0.47 | 0.45 | Dominant | 1.05 | 0.65-1.68 | 0.848 |  |
|  |  |  |  |  |  | Recessive | 1.29 | 0.76-2.19 | 0.339 |  |
|  |  |  |  |  |  | Additive | 1.11 | 0.81-1.53 | 0.499 |  |
|  |  |  |  |  |  | Allelic |  |  | 0.603 | 0.27 |
|  |  |  |  |  |  |  |  |  |  |  |
| rs3212986 | 19 | 45912736 | A | 0.24 | 0.21 | Dominant | 1.17 | 0.76-1.79 | 0.47 |  |
|  |  |  |  |  |  | Recessive | 1.39 | 0.60-3.19 | 0.440 |  |
|  |  |  |  |  |  | Additive | 1.17 | 0.83-1.64 | 0.377 |  |
|  |  |  |  |  |  | Allelic |  |  | 0.424 | 0.64 |
|  |  |  |  |  |  |  |  |  |  |  |
| rs20579 | 19 | 48668830 | A | 0.14 | 0.13 | Dominant | 1.15 | 0.71-1.85 | 0.565 |  |
|  |  |  |  |  |  | Recessive | 0.83 | 0.09-7.35 | 0.865 |  |
|  |  |  |  |  |  | Additive | 1.12 | 0.72-1.75 | 0.616 |  |
|  |  |  |  |  |  | Allelic |  |  | 0.659 | 0.19 |
|  |  |  |  |  |  |  |  |  |  |  |
| rs132771 | 22 | 42025350 | A | 0.16 | 0.16 | Dominant | 0.99 | 0.63-1.58 | 0.990 |  |
|  |  |  |  |  |  | Recessive | 0.83 | 0.05-3.33 | 0.408 |  |
|  |  |  |  |  |  | Additive | 0.95 | 0.62-1.45 | 0.809 |  |
|  |  |  |  |  |  | Allelic |  |  | 0.899 | 0.02 |

***a***: MAF=Minor Allele Frequency ***b*:** OR adjusted for age, sex, and country
